# Anti-Hemagglutinin Antibody Derived Lead Peptides for Inhibitors of Influenza Virus Binding

**DOI:** 10.1371/journal.pone.0159074

**Published:** 2016-07-14

**Authors:** Henry Memczak, Daniel Lauster, Parimal Kar, Santiago Di Lella, Rudolf Volkmer, Volker Knecht, Andreas Herrmann, Eva Ehrentreich-Förster, Frank F. Bier, Walter F. M. Stöcklein

**Affiliations:** 1 Department of Bioanalytics and Biosensorics, Fraunhofer Institute for Cell Therapy and Immunology, Branch Bioanalytics and Bioprocesses, Potsdam, Germany; 2 Institute of Biochemistry and Biology, University of Potsdam, Potsdam, Germany; 3 Department of Biology, Humboldt Universität zu Berlin, Berlin, Germany; 4 Department of Theory and Bio-Systems, Max-Planck-Institute of Colloids and Interfaces, Potsdam, Germany; 5 Departamento de Química Biológica e IQUIBICEN-CONICET, Facultad de Ciencias Exactas y Naturales, Universidad de Buenos Aires, Buenos Aires, Argentina; 6 Institute of Immunology, Charité, Berlin, Germany; 7 Department of Biosystem Integration and Automation, Fraunhofer Institute for Cell Therapy and Immunology, Branch Bioanalytics and Bioprocesses, Potsdam, Germany; Icahn School of Medicine at Mount Sinai, UNITED STATES

## Abstract

Antibodies against spike proteins of influenza are used as a tool for characterization of viruses and therapeutic approaches. However, development, production and quality control of antibodies is expensive and time consuming. To circumvent these difficulties, three peptides were derived from complementarity determining regions of an antibody heavy chain against influenza A spike glycoprotein. Their binding properties were studied experimentally, and by molecular dynamics simulations. Two peptide candidates showed binding to influenza A/Aichi/2/68 H3N2. One of them, termed PeB, with the highest affinity prevented binding to and infection of target cells in the micromolar region without any cytotoxic effect. PeB matches best the conserved receptor binding site of hemagglutinin. PeB bound also to other medical relevant influenza strains, such as human-pathogenic A/California/7/2009 H1N1, and avian-pathogenic A/Mute Swan/Rostock/R901/2006 H7N1. Strategies to improve the affinity and to adapt specificity are discussed and exemplified by a double amino acid substituted peptide, obtained by substitutional analysis. The peptides and their derivatives are of great potential for drug development as well as biosensing.

## Introduction

Influenza A virus is an enveloped virus belonging to the Orthomyxoviridae family. It can cause annual epidemics and infrequent pandemics [1]. The Spanish flu pandemic of 1918 as well as the Asian flu of 1957 and the Hongkong flu in 1968 pandemics caused the death of millions of people [2]. In 2009 the pandemic swine origin influenza A H1N1 virus as well as the outbreak of H7N9 in China in 2013 has reminded the world of the threat of pandemic influenza [3–6].

The genome of influenza virus consists of eight segmented negative RNA strands. The envelope bilayer harbors the two spike glycoproteins hemagglutinin (HA) and neuraminidase (NA), and the M2 proton channel. The homotrimeric HA is the most abundant protein on the viral surface. It mediates attachment to the host cell surface via binding to sialic acid (SA) residues of cellular receptors, and upon endocytic virus uptake it triggers fusion of the envelope with the endosomal membrane releasing the viral genome into the cytoplasm. NA cleaves glycosidic bonds with terminal SA facilitating the release of budding virions from the cell.

In diagnostics, antibodies against spike proteins are the preferred tool for identification and serotyping of viruses. Development of therapeutic antibodies against influenza is a challenge, as the high viral mutation rate (antigenic drift) and genetic reassortment of the virus genome (antigenic shift) continuously lead to new strains escaping from neutralization by antibodies [7, 8]. This goes along with adaptation to small molecule inhibitors (e.g. oseltamivir) [9].

Vaccines can only temporarily control the recurring epidemics of influenza, because antigenic changes are typical for HA and NA. 16 avian and 2 bat serotypes of influenza A virus HA (H1—H18) are known, but only three (H1, H2, and H3) have been adapted to humans. Antibodies binding to regions of hemagglutinin conserved among serotypes have been developed which demonstrated broad specificity and neutralization potency [10–15]. However, development, production and quality control of antibodies is expensive and time consuming.

As an alternative, short peptides binding specifically to the spike proteins can be produced in automated high-throughput synthesis at low costs. HA-binding peptides have been recently obtained by phage display, lead structure optimization of natural products and specific toxins, bioinformatics tools and discovery from side effects of known anti-inflammatory peptides [16–23]. Some of them showed antiviral activity [17, 19–23]. A more epitope-oriented accession to binding peptides is the search for paratope-derived peptides from variable regions of specific antibodies [24]. Antibodies against HA have been described, and at least 6 antigenic sites (A-F) on the HA-trimer have been identified, localized either at the receptor binding site, the interface of the three HA-monomers, or at other sites like the stalk [8, 11, 25]. Several structures of HA–antibody complexes have been published deposited in the protein data bank (PDB) [11–14]. Indeed, an antibody was described, whose HA binding is mediated mainly by one CDR, namely HCDR3 [12].

Inspired by this finding, we chose linear peptides corresponding to the CDRs of VH of monoclonal antibody HC19, having the majority of contacts with the HA1 domain of the strain A/Aichi/2/1968 [26, 27]. The antibody and the derived peptides bind to HA at the SA binding site, in particular to the 130-loop and the 190-helix, which belong to the antigenic sites A and B, respectively. This binding site is conserved among several HA serotypes providing a basis for a peptide with broader specificity [28].

We used complementary experimental and theoretical approaches to select HA binding VH-CDR peptides and to improve their potential to inhibit binding, and finally, infection of cells by influenza A virus. The inhibitory potential of the most efficient CDR-peptide was improved by microarray-based site-directed substitutions of amino acids. We could demonstrate a broader specificity of the selected peptides as they bound to HA of human and avian pathogenic influenza strains.

## Material and Methods

### Virus material

Influenza strain A/Aichi/2/68 H3N2 X31 (Aichi H3N2), reassorted with A/PuertoRico/8/1934 H1N1, and low pathogenic A/Mute Swan/Rostock/R901/2006 H7N1 K3141 (Rostock H7N1) were harvested from allantoic fluid of hen eggs. Virus isolates were clarified upon low speed centrifugation (300 x g, 10 min) and concentrated by ultracentrifugation (100 000 x g, 1 h). For safety reasons, viruses prepared for SPR experiments were inactivated by 5 min irradiation with UV-light on ice. Infectivity of inactivated virus was precluded using MDCK II based cell-assays, remaining binding ability of HA was proven using standard hemagglutination assay (HA) with human red blood cells [29]. For SPR measurements, also monovalent split vaccine influenza strains A/NewYork/55/2004 H3N2, NIH accession No. ABO37541 (New York H3N2), A/Victoria/210/2009 H3N2, NIH accession No. AFM71802 (Victoria H3N2) and the split vaccine Pandemrix (GlaxoSmithKline) of influenza strain A/California/7/2009 H1N1, NIH accession No. ACP44189 (California H1N1) were used. Protein concentrations were determined using standard BCA assay (Thermo Fisher Scientific). Additionally viruses were titrated for HA units. Here, 50 μl virus concentrate was serially diluted twofold in PBS using 96-well microtiter plates. Then, 50 μl of 1% human red blood cells (German Red Cross, Berlin, AB+) were added to each well, followed by an incubation of 60 min at room temperature. The last well showing hemagglutination provided the HA units per 50 μl virus solution. For the Rostock H7N1 strain turkey erythrocytes have been used. Additional material used was HA from Aichi H3N2 (Sino Biological, 11707-V08H).

### Cell lines

For infection and cytotoxicity assays Madin-Darby Canine Kidney Epithelial Cells (MDCK II, NBL-2, CCL-34) were used (ATCC). Cells were cultivated under standard cell culture conditions with DMEM (supplemented with 10% fetal calf serum (FCS), 2 mM L-glutamine) in humid atmosphere at 37°C and 5% CO_2_. Hemagglutination inhibition assays (HAI) were performed using either human erythrocytes (α-2,6’-sialosugars) for Aichi H3N2, or turkey erythrocytes (α-2,3’-sialosugars) for Rostock H7N1 [29].

### Peptides

Peptides were synthesized with a linker for immobilization or without linker for *in vitro* inhibition assays. For SPR based binding experiments, antibody derived peptides were extended with an N-terminal lysine linker: KKKK-SGFLLISN-amide (PeA-Lys), KKKK-FYDYDVFY-amide (PeB-Lys), KK-ßAßA-LGVIWAGGNTNY-amide (PeC-Lys). In the SPR based screen for virus binding, single and double mutated variants of PeB-Lys were used. All lysine variants were purchased from Biosyntan or Genecust with >95% purity. These peptides were dissolved in water and diluted in appropriate buffers for immobilization on SPR sensor chips. SPR based binding inhibition assays were performed with the full length peptides SGFLLISNGVHWV-amide (PeA), ARDFYDYDVFYYAMD-amide (PeB), and its double mutant ARDFYGYDVFFYAMD-amide (PeB^GF^). For the biological assays PeB, PeB^GF^ and the control peptide ARDFYDPDVFYYAMD-amide (PeB^P^) were applied. These peptides, and the peptides EB, s2(1–5), Phage 1 (P1) H5N1 and Phage 1 (L-P1) H9N2, Mucroporin M-1were synthesized by Rudolf Volkmer (Charité, Berlin). HPLC purification and analysis were achieved using a linear solvent gradient (A: 0.05% TFA in water; B: 0.05% TFA in acetonitrile; gradient: 5–60% B over 30 min; UV detector at 214 nm; RP-18 column). The identities of the peptides were validated by mass spectrometry using MALDI-TOF (microflex LT, Bruker Daltonik), and ESI (Q-TOF micro, Micromass). Lyophilized peptides could be stored for at least one year at -20°C as controlled by HPLC/MS analysis. Stock solutions of PeB, PeB^GF^ and PeB^P^ were prepared by initial dissolving in DMSO, followed by dilution in PBS (8 mM peptide with 10% DMSO v/v). Solvation was improved by sonication. Stock solutions could be stored for several weeks at 4°C.

Circular Dichroism (CD) spectroscopy for determination of secondary structure and melting temperature was performed (S1 Fig) using a Jasco J-715 CD spectrometer. CD spectra of 100 μg ml^-1^ peptide solutions were recorded at 1 nm wavelength steps. The primary signal in millidegrees (mdeg) was recorded over 4 s, and then the molar ellipticity (θmrw, [deg cm^2^ dmol^-1^ 10^−3^]) was calculated.

### Surface preparation for surface plasmon resonance

Measurements were performed on a Biacore™ T200 (GE-Healthcare Bio-Sciences AB) using CM5 chips, and running buffer HBSP (10 mM Hepes, 150 mM NaCl, 0.05% Tween 20, pH 7.4) at 25°C. Peptides and proteins were immobilized via amine coupling with EDC/NHS at a flow rate of 10 μl min^-1^, according to the manufacturer’s instructions (GE Healthcare). Optimum preconcentration was evaluated before, using acetate and phosphate buffers (10 mM) of pH 4 to 7, and ligand concentrations of 10 μg ml^-1^. For the immobilization of biotinylated sialyllactose (Lectinity), neutravidin (Thermo Fisher Scientific) (100 μg ml^-1^) was immobilized by amine coupling at pH 5.5 enabling high ligand density. Biotinylated sialyllactose was then injected at a concentration of 10 μg ml^-1^ in HBSP for 3 min.

### Binding experiments

Several concentrations of analyte (HA, virus) were applied at least onto two channels with generally 500 s association and a 600 s dissociation time, followed by a 60 s regeneration step with 50 mM NaOH. A flow rate of 10 μl min^-1^ was used. Quantitative data were obtained by extracting the amount of bound material 10 s after injection was terminated (mean value over 5 s). Reverse peptide binding experiments were run, with immobilized HA or Aichi H3N2, under the same conditions. Double referencing of binding curves was performed, using a control flow cell without ligand, and using control injections of buffer, in order to eliminate bulk refractive index effects and unspecific binding to the sensor surface. The dissociation constant (*K*_*D*_) was calculated from a plot of steady state binding levels against analyte concentration, using a Biacore T200 Evaluation Software.

### Solid-state binding competition experiments

A constant concentration of 50 or 100 μg ml^-1^ Aichi H3N2 virus was used as analyte, while the concentration of the inhibitors was varied. The suspensions of virus and inhibitor were mixed and subsequently incubated on a Thriller® thermoshaker at 500 rpm and 37°C for at least 15 min to reach equilibrium before injection on the SPR surface. The inhibitors used were peptides or α-2,3’- and α-2,6’-sialyllactose (Carbosynth). The suspensions of virus and inhibitor were mixed and subsequently incubated for at least 15 min to reach equilibrium before injection on the SPR surface. All data were referenced against buffer or inhibitor injection of identical concentration. The decrease of binding capacity of the chip over time was considered by referencing against multiple virus injections at various time points during the experiment. Inhibition was calculated against the mean value of at least five binding events with the virus alone, using the sigmoidal four parameter logistic fit (Origin).

### Microarray based substitutional analysis

Microarray-based substitutional analysis of peptide PeB was performed using a PepStar^®^ peptide library spotted on glass slides by JPT Peptide Technologies. The slides were used without additional treatment. For the labeling of proteins with a fluorescent dye, Dyomics DY-634 (λ_ex_ = 635 nm, λ_em_ = 654 nm, Fluorospin 634 Kit (emp Biotech) was used, according to the manufacturer´s instructions. The following materials were labeled: NewYork H3N2, Victoria H3N2, Aichi H3N2, and California H1N1. Labeled analytes were incubated several hours or overnight at indicated concentrations using Femtotip buffer (FTP) (20 mM Tris, 30% glycerol, 3% polyvinylpyrrolidon 90, 0.1% Tween 20, pH 8.4) for dilution. The slides were washed twice in FTP and twice in ultrapure water and subsequently dried under a stream of nitrogen. Fluorescence measurements were performed using an Axon 4200A Laser Scanner (Molecular Devices). Fluorescence intensity was evaluated using GenePix Pro 6.0 software. For evaluation of binding efficiency the contrast (*C*) was calculated from

$$C=\frac{{\text{I}}_{\text{S}}\;\text{-}\;{\text{I}}_{\text{NC}}}{{\text{I}}_{\text{S}}+{\text{I}}_{\text{NC}}}$$

where *I*_S_ represents the relative fluorescence intensity (background subtracted) of a sample spot and *I*_NC_ the relative fluorescence intensity of an appropriate negative control spot. Mean values of triplicate experiments for mutant peptides (*C*_*M*_) are given relative to contrast of positive control fetuin (*C*_M_ x 100/*C*_fetuin_) in percent with *C*_*PeB*_ set to zero. Experimental data can be obtained from GEO accession GSE78700, ID 200078700.

### Neuraminidase activity assay

The assay was performed according to Potier et al. [30]. Recombinant neuraminidase of Influenza A virus (A/Aichi/2/1968, H3N2) was purchased from antibodies-online (ABIN2007091). The substrate 4-Methylumbelliferyl-N-acetyl-α-D-neuraminic acid sodium salt (MUNANA) was obtained from Carbosynth. The assays were done in MES buffer, containing 32.5 mM 2-(N-morpholino-) ethane sulfonic acid and 4 mM CaCl_2_. The fluorescence of the cleaved product 5-Methyl-umbelliferone was detected by the FLUOstar Omega Microplate Reader (BMG LABTECH, Ortenberg, Germany) at excitation wavelength 320 nm and emission wavelength 510 nm. The well-known inhibitor N-acetyl-2,3-dehydro-2-deoxy- neuraminic acid disodium salt (DANA, Carbosynth) was used as a positive control for the inhibition assay. The microtiter plate wells were filled with 50 μl containing 15 ng neuraminidase, 200 μM substrate and various concentrations of inhibitor, and incubated at 37°C for 30 min. Assays were also performed with Aichi H3N2, UV inactivated, 100 ng per well.

### Hemagglutination inhibition assay

Aichi H3N2 or Rostock H7N1 viruses were incubated with human or turkey erythrocytes, respectively, to yield agglutination (in the absence of an inhibitor) and concentration-dependent inhibition of agglutination (in the presence of an inhibitor). Inhibitors were twofold serially diluted in PBS. Then, 2 hemagglutination units (HAU) containing 2∙10^7^ virus particles were added to all wells. Viral particle concentration was estimated as described by Desselberger et al. [31]. After 30 min incubation at room temperature, 50 μl of a 1% erythrocyte solution (~2∙10^6^ cells μl^-1^) was added, gently mixed and incubated for 60 min at room temperature. For Aichi H3N2 human erythrocytes (α-2,6’-sialosugars), for Rostock H7N1 turkey erythrocytes (α-2,3’-sialosugars) have been used. The inhibitor constant *K*_*i*_(HAI), reflects the lowest inhibitor concentration, which is necessary to achieve complete inhibition of hemagglutination caused by the influenza virus. To check for full hemagglutination inhibition, the microtiter plate was tilted by 60° to cause droplet formation from the red blood cell pellet [29].

### Infection inhibition assay

The experiment has been assessed using an MTS reagent (Promega) according to the manufacturer’s protocol: 15,000 MDCK II cells were seeded the day before infection. Aichi H3N2 or Rostock H7N1 were pretreated with peptides in a twofold dilution series for 30 min at room temperature under slight agitation. Cells were washed once with PBS (supplemented with 0.49 mM Mg^2+^, 0.90 mM Ca^2+^), before pretreated virus (MOI 0.05) was added to the cells and maintained for 1 h at room temperature to allow binding. Unbound virus was removed by washing once with infection medium (DMEM, 2 mM L-glutamine, 0.1% FCS, 0.1% bovine serum albumin (BSA), 2.5 μg ml^-1^ L-(tosylamido-2-phenyl) ethyl chloromethyl ketone (TPCK) treated trypsin, 100 U ml^-1^ penicillin and 100 μg ml^-1^ streptomycin), and subsequently incubated for 24 h at 37°C. Then, 20 μl MTS solution was added to each well followed by 2 hours incubation at 37°C. Finally, absorbance at 490 nm was recorded and data were normalized as follows:

$$\text{Infection inhibition}\;(\%)=\frac{\left({\text{Infected}}_{\text{treated}}\text{-}{\text{Infected}}_{\text{untreated}}\right)}{\left({\text{Uninfected}}_{\text{untreated}}\text{-}{\text{Infected}}_{\text{untreated}}\right)}\text{x}\;100$$

Experiments have been performed for both virus strains in at least triplicate experiments.

### Microneutralization assay

The microneutralization assay was performed according to the protocol of Klimov et al. [32] with minor modifications. Briefly, peptide dilutions were mixed with either 200 TCID_50_ Aichi H3N2 or 50 TCID_50_ Rostock H7N1 in infection medium for 30 min at room temperature. Subsequently, 30,000 MDCK II cells were added to each well, followed by incubation for 18 h at 37°C. Next, the medium was removed, washed with PBS and fixed with ice cold acetone (80% v/v). After removal of the fixative, plates were dried and undergone immunostaining. Cells were stained with IgG primary antibody against the influenza A nucleoprotein (Millipore, Cat. # MAB8257, mouse monoclonal, 1:1000 in antibody diluent: PBS, 0.3% Tween 20 (v/v) and 5% (w/v) nonfat, dry milk) for 1 h at room temperature. Following, cells were washed with washing buffer (PBS, 0.3% Tween 20), before a secondary antibody (1:1000 goat anti-mouse IgG, KPL, Cat. # 074–1802 in antibody diluent) conjugated to horseradish peroxidase (HRP) was added for another hour at room temperature. Next, the antibody was removed and the cells were washed with washing buffer. Finally, σ-phenylenediamine dihydrochloride (OPD) in citrate (0.05 M phosphate-citrate, 0.03% sodium perborate, pH 5.0 at 25°C), was added for approximately 30 minutes until the supernatant turned yellow. The reaction was stopped with 0.5 M sulfuric acid and absorbance of the reaction product in the microwell plate was recorded at 490 nm. After subtraction of the background of non-infected cells, neutralization was calculated in ratio to untreated, infected cells.

### Cell viability assay

One day before treatment 15,000 MDCK II cells were seeded. On the following day media was removed and replaced with a twofold serial dilution of either PeB, PeB^GF^ or PeB^P^ in supplemented DMEM (see above). Next, cells have been incubated in the presence of inhibitor for 24 h at 37°C. Then, 20 μl MTS solution was added to each well. Finally, absorbance at 490 nm was measured and data were normalized to untreated cells as follows:

$$\text{Cell viability}\;(\%)=\frac{\left(\text{Treated Cells}\;\text{-}\;\text{Medium Background}\right)}{\left(\text{Untreated Cells}\;\text{-}\;\text{Medium Background}\right)}\text{x}\;100$$

### Molecular dynamics simulations

An ensemble of configurations for the complexes HA-PeA, HA-PeB, HA-PeC and HA-PeB^GF^ was built using the AMBER14 package of programs [33]. Original coordinates for all atoms were taken from crystal structure PDBid = 2VIR. In each case, the antibody moiety excepting the sequence of the peptide under study was erased. In the case of PeB^GF^, *in silico* D6G and Y11F mutations were performed. Systems were then solvated using the TIP3P water molecule model [34], extending at least 10 Å from the complex. Nearly 18250 water molecules were added to solvate the complex and the resulting truncated octahedral box size was nearly 108 Å x 108 Å x 108 Å. An appropriate number of chloride ions were added to keep the total system charge neutral. All bond lengths involving hydrogen atoms were constrained using the SHAKE algorithm allowing the usage of a 2 fs time-step [35]. The temperature was fixed at 300 K using a Langevin thermostat with a collision frequency of 2 ps^-1^. The electrostatic interactions were treated using the particle-mesh Ewald (PME) scheme with a fourth-order B-spline interpolation and a tolerance of 10^-5^ [36]. The non-bonded cut-off was 8 Å and the non-bonded pair list was updated every 50 fs.

Simulations were carried out according to the same protocol that has been used in our previous studies [37–41], with the Amber FF99SB force field [42]. Briefly, each complex configuration was first optimized by 1000 steps of steepest descent followed by another 1000 steps of conjugate gradient minimization, keeping all atoms of the complex restrained to their initial position with a weak harmonic potential. Each system was then simulated for 50 ps at constant volume with a 2 kcal mol^-1^ Å^-2^ restraint on the complex, in order to equilibrate the solvent at 300 K without undesirable drifts of the structure. Subsequently, a 50 ps MD simulation with a 2 kcal mol^-1^ Å^-2^ restraint on each complex at a pressure of 101,325 kPa was conducted to relax the density using Berendsen's barostat. After 1 ns without restraint equilibration phase, a 10 ns simulation at constant pressure was carried out and the coordinates were stored every 10 ps, resulting in 1000 configurations for each simulation. Subsequently, Molecular Mechanics-Poisson-Boltzmann Surface Area (MM-PBSA) calculations were performed.

### Molecular Mechanics—Poisson-Boltzmann Surface Area calculations

In the MM-PBSA method, the binding free energy of the receptor-ligand complex, ΔG_bind_, is determined from

$$\Delta {\text{G}}_{\text{bind}}={\text{G}}_{\text{com}}\;\text{-}\;{\text{G}}_{\text{rec}}\;\text{-}\;{\text{G}}_{\text{lig}}$$

where G_com_, G_rec_, and G_lig_ denote the absolute free energies of the complex, receptor and the ligand, respectively. This method has been discussed elsewhere [37–41].

The free energy G for each species is estimated from

$$\text{G}={\text{E}}_{\text{MM}}\;\text{-}\;{\text{G}}_{\text{solv}}\;\text{-}\;{\text{T}}_{\text{MM}}\text{S}$$

Here, E_MM_ is the molecular mechanical energy in the gas phase, G_solv_ the solvation free energy, and -TS_MM_ the contribution from the conformational entropy. The term E_MM_ is comprised of the internal (bond, angle, dihedral) (E_int_), electrostatic (E_elec_), and van der Waals energies (E_vdW_), according to

$${\text{E}}_{\text{MM}}={\text{E}}_{\text{int}}+{\text{E}}_{\text{elec}}+{\text{E}}_{\text{vdW}}$$

and

$${\text{E}}_{\text{int}}={\text{E}}_{\text{bond}}+{\text{E}}_{\text{angle}}+{\text{E}}_{\text{torsion}}$$

To incorporate all possible nonbonded interactions, the term *E*_MM_ was estimated for each snapshot with no cut-offs. The solvation free energy, *G*_solv_, is approximated as the sum of the polar (*G*_pol_) and the nonpolar contribution (*G*_np_) using a continuum representation of the solvent according to

$${\text{G}}_{\text{solv}}={\text{G}}_{\text{pol}}+{\text{G}}_{\text{np}}$$

$${\text{G}}_{\text{np}}=\gamma \;\text{x}\;\text{SASA}+\text{b}$$

Here, *γ* = 0.00378 kcal mol^-1^ Å^-2^ and *b* = -0.5692 kcal mol^-1^. The popular linear Poisson-Boltzmann method was used to estimate the polar component of the solvation free energy. Here, SASA is the solvent accessible surface area estimated by the linear combination of pairwise overlap (LCPO) algorithm using a probe radius of 1.4 Å [43].

The electrostatic contribution to the solvation free energy was estimated from the Poisson-Boltzmann (PB) approach using the *pbsa* solver implemented in Amber. In order to solve the PB equation the grid spacing was set to 0.5 Å in all dimensions and the relative dielectric constants in the protein and in the water were chosen to be 1 and 80, respectively. The ionic strength was set to 0.15 M. The ratio between the longest dimension of the rectangular finite-difference grid and that of the solute was chosen to be 4.0. The linear PB equation was solved with a maximum of 1000 iterations.

In order to understand the inhibitor-residue interaction in more detail, the interaction energy was further decomposed into the contributions from each residue of the peptide by using the theory of free energy decomposition [44]. The peptide-residue interaction is approximated by

$${\Delta \text{G}}_{\text{peptide-residue}}={\Delta \text{E}}_{\text{vdw}}+{\Delta \text{E}}_{\text{elec}}+{\Delta \text{G}}_{\text{PB}}$$

where ΔE_vdW_ and ΔE_elec_ are the contributions from the van der Waals and electrostatic interactions between the inhibitor and each residue in the gas phase. The polar solvation free energy, ΔG_PB_, was estimated using the *pbsa* module of Amber.

## Results

### Modeling based selection of HA binding peptides

**Fig 1:**
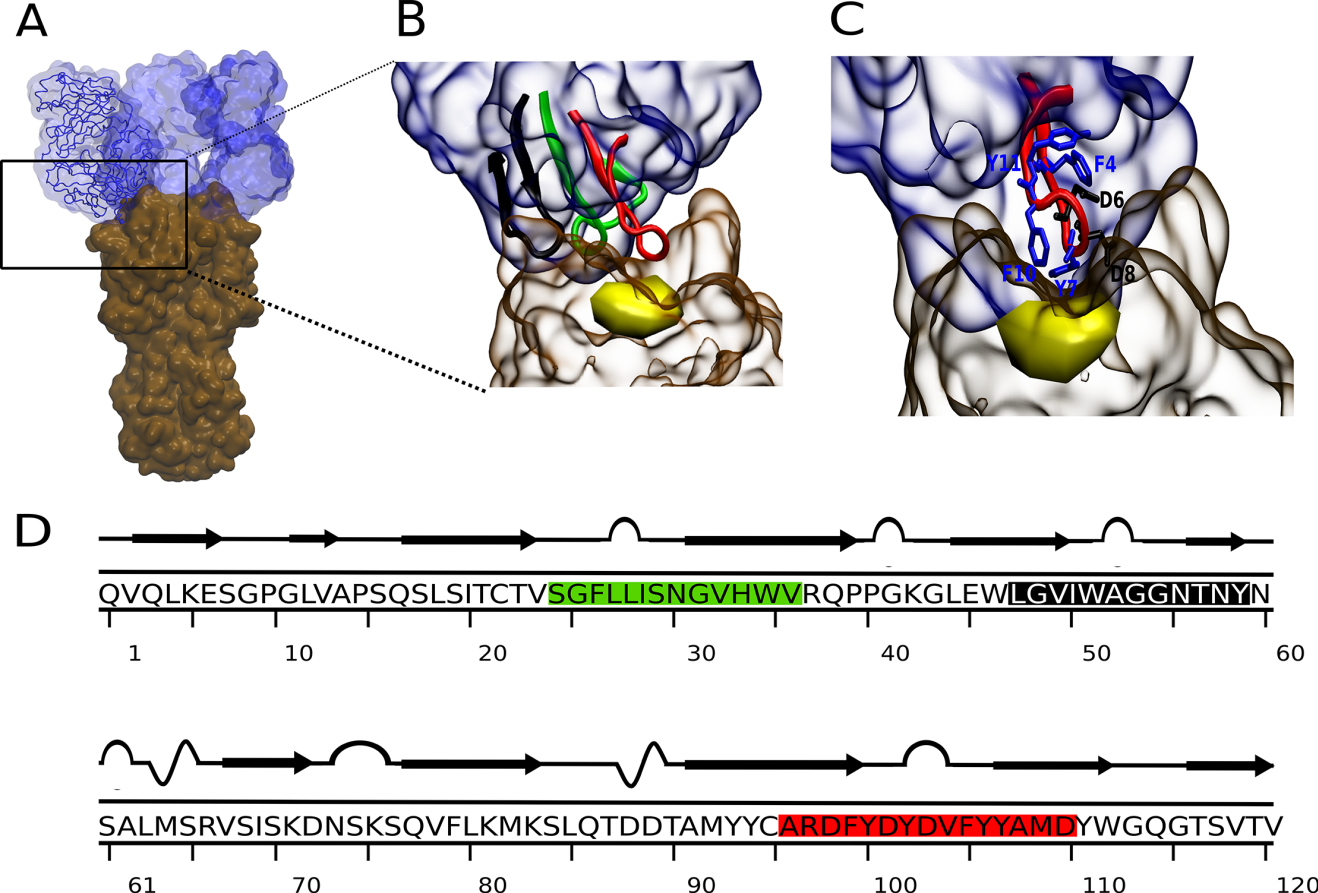
Structure of the HC19 antibody in complex with the HA1 domain of Aichi H3N2. A) 3D structure three fragments of antibody (Fab, blue) bound to the HA1 domains of trimeric HA (brown) of Aichi H3N2 (PDB: 2vir) [26]. B) Zoom in on the CDR containing Fab subdomain. Peptides containing the CDR sequences 26–32 (CDR1), 53–55 (CDR2) and 99–110 (CDR3) were entitled PeA (green), PeC (black) and PeB (red) [27]. C) PeB interacting with HA in proximity to the sialic acid binding pocket (yellow). Amino acids beneficial for binding are shown in blue, non-favorable amino acids (see **Results**) are shown in black. D) Primary and secondary structures of VH of the Fab fragment. Antibody derived peptides are colored as in B.

The 3D structure of the Fab-hemagglutinin complex of the antibody HC19 (PDB: 2vir; complex of immunoglobulin IgG1 with HA of Aichi H3N2) was analyzed to identify peptide sequences of the Fab fragment at the HA contact area [26]. Essentially, three hypervariable complementarity determining regions (CDR) of the heavy chain variable domain (VH) of Fab interact with HA (Fig 1A, 1B and 1C). From these three loop-like organized sequences interacting with the SA binding pocket of HA, peptides PeA, PeB and PeC (Fig 1D) were derived containing as a core the CDR sequences (Table 1).

**Table 1:** Total free energy of binding of CDR-related peptides to HA of Aichi H3N2. CDR sequences are underlined.

Peptide	Sequence	Δ*G* [kcal mol^-1^]
PeA	SGFLLISNGVHWV	*dissociation*
PeB	ARDFYDYDVFYYAMD	-20.4
PeC	LGVIWAGGNTNY	-5.3

**Fig 2:**
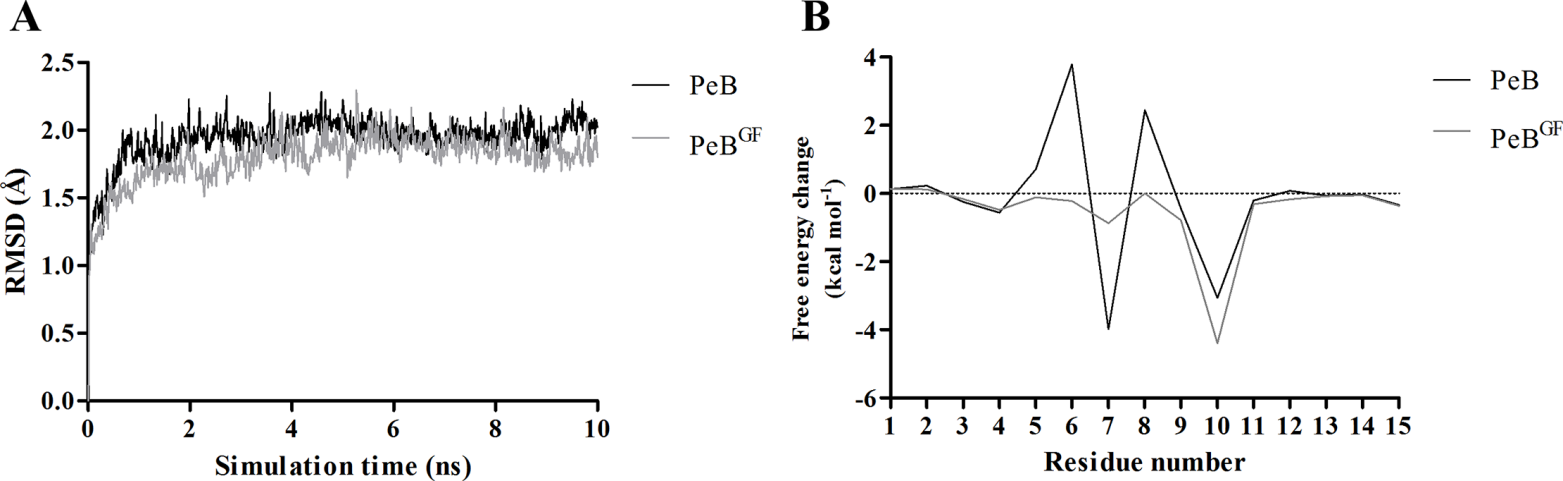
MD simulation of peptide-hemagglutinin complexes. A) Time evolution of root-mean-square deviations (RMSDs) of backbone atoms relative to their initial configurations for Aichi H3N2 HA complexed with PeB (black) and PeB^GF^ (gray). N- and C-terminal flexible tails were excluded for RMSD calculation. B) Contributions of the individual residues of PeB (black) and PeB^GF^ (gray) to the total binding free energy change.

In order to predict and compare binding affinities to HA of the three peptides, corresponding peptide-HA complexes were simulated in explicit water model for 10 ns using MD. As deduced from the root-mean-square deviation for PeB already after three ns a steady state for binding was observed (Fig 2A). In contrast, peptide PeA dissociated from HA within the simulation time of 10 ns. Gibbs free energies of peptide binding to HA were -20.4 and -5.3 kcal mol^-1^ for PeB and PeC, respectively (Table 1). Even though absolute values for free energy changes derived from MD simulation have been proven not to correlate exactly with experimental values, a comparison between MD derived values implicates a stronger binding of PeB to HA. Also, as shown in Fig 1B and 1C, PeB fits best the conserved SA binding site of Aichi H3N2 HA. Based on this and experimental results (see below) we selected PeB as a potential inhibitor of virus binding to host cells. As expected for its short primary sequence, and revealed experimentally by CD spectroscopy, PeB displayed no secondary structure elements in solution at physiological pH (S1A and S1B Fig).

To unravel the molecular basis of PeB binding to HA, an analysis of energetic contribution was conducted by a combined MD/MM-PBSA approach. Molecular configurations obtained from MD simulations of the complexes in explicit water were used for calculation of binding free energies using an implicit solvation scheme. The binding free energy was decomposed into contributions of the individual peptide residues (Fig 2B, PeB). Residues within and close to the loop (Y5 to Y12) provide the largest contributions to the binding free energy. Significant attractive contributions to the binding free energy change arise from F4 (-0.6 kcal mol^-1^), Y7 (-3.9 kcal mol^-1^), and F10 (-3.0 kcal mol^-1^). However, D8 and D6 contribute repulsively by 2.4 kcal mol^-1^ and 3.7 kcal mol^-1^, respectively, to the binding free energy. Hence, exchanging D8 and D6 to other amino acids would predict to reduce the repulsive interactions of these residues and, thus, to increase the affinity of the modified PeB to HA. The detailed analysis of electrostatic, van der Waals and polar contributions to the free energy change of each residue is presented in S3 Fig.

We have also analyzed the contributions of the individual residues of HA to this complex. Attractive contributions mainly originate from residues S136 (-3.0 kcal mol^-1^), N137 (-1.8 kcal mol^-1^), and L194 (-1.0 kcal mol^-1^), whereas the most significant repulsive contribution is caused by E190 (1.1 kcal mol^-1^) (numbering according to PDB 2vir) (S4 Fig). The attractive contributions from the polar residues S136 and N137 arise mainly from the intermolecular electrostatic interaction energy, whereas the hydrophobic residue L194 derives most of its contribution from van der Waals interactions.

### Binding of peptides to hemagglutinin

**Fig 3:**
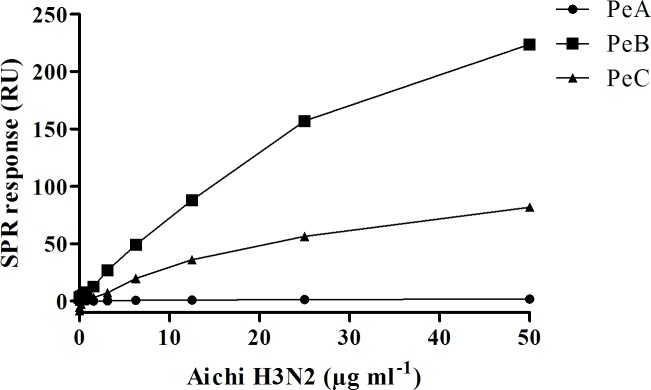
Binding of influenza virus to antibody derived peptides. Aichi H3N2 virus was injected on surface immobilized peptides. PeA-Lys (0.77 pmol mm^-^²), PeB-Lys (0.57 pmol mm^-^²) and PeC-Lys (1.93 pmol mm^-^²). The SPR signal at 10 s after terminating injection was taken as binding response.

Binding of Aichi H3N2 virus to surface immobilized peptides was measured using SPR. For this purpose, peptides PeA-Lys, PeB-Lys, and PeC-Lys, containing lysine residues at the amino terminus, acting as spacer were used. As deduced from MD simulations, the N-terminus of PeB does not significantly contribute to binding of the peptide to HA, as the contribution of these residues to the total free energy change is close to 0 kcal mol^-1^ (Fig 2B, PeB). Binding responses are shown in Fig 3, deduced from the SPR sensorgrams for the binding of Aichi H3N2 to peptide-covered surfaces (S5 Fig).

**Fig 4:**
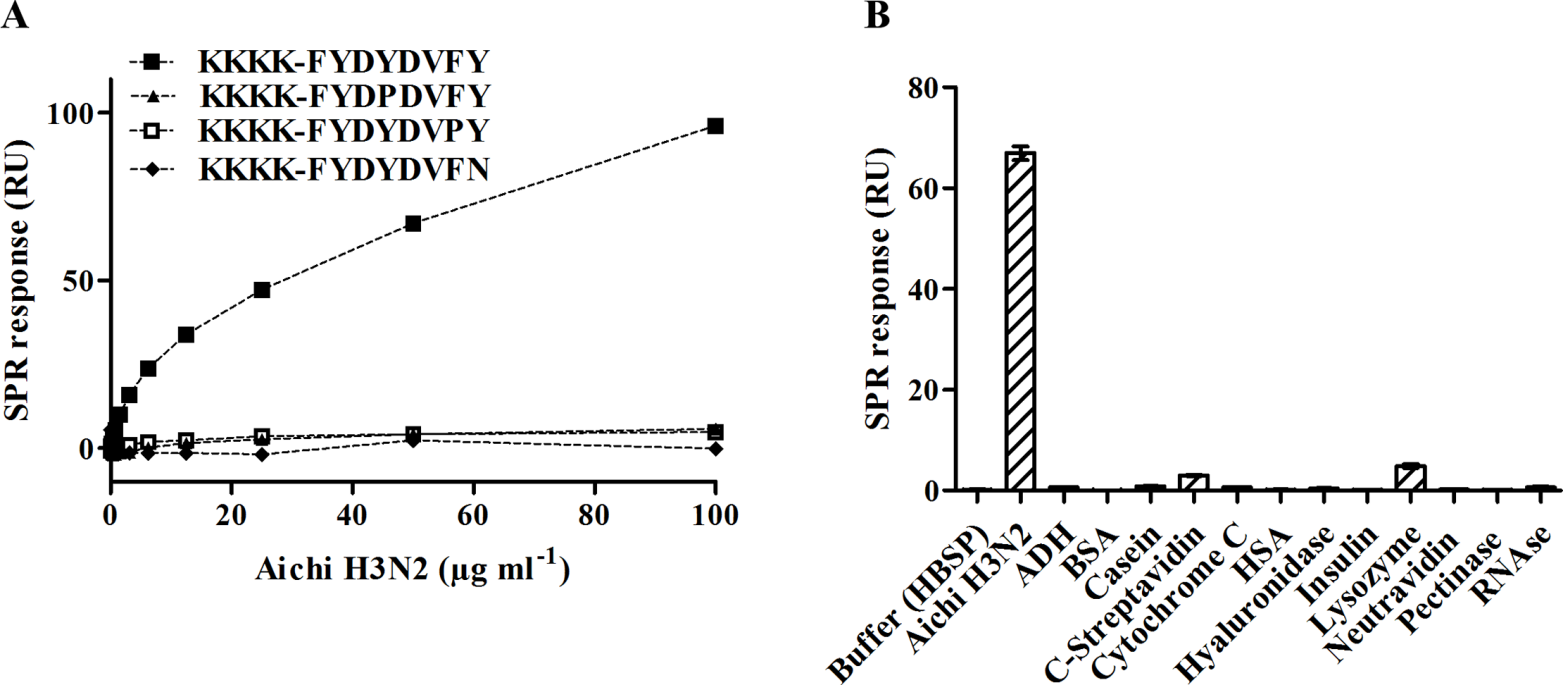
Specificity of virus-peptide binding. A) Dose-response curves for binding of Aichi H3N2 to immobilized PeB-Lys and three monosubstituted derivatives of the peptide. B) Binding of Aichi H3N2 virus to immobilized PeB-Lys compared with a set of well-known proteins (S1 Table). All proteins were diluted to a concentration of 10 μg ml^-1^. Columns represent the mean value of duplicate experiments. The error bars show the SEM.

Significant virus binding was observed on PeB- and PeC-, but not on PeA-modified surfaces consistent with our MD simulations. The minimum detection level, corresponding to the lowest virus concentration, and showing binding 3 times above the SPR response of a buffer control, was 0.19 μg and 6.25 μg Aichi H3N2 virus protein ml^-1^ for immobilized PeB and PeC, respectively. The higher sensitivity of PeB in the viral binding assay confirms the selection of this CDR derived peptide for further experiments, as mentioned above. To confirm specificity three PeB variants containing single amino acid residue exchanges, which were immobilized with the same ligand density, were investigated. No binding was observed with the three variants, suggesting that the binding of virus is indeed specific to the CDR sequence (Fig 4A). The results also exclude the possibility that binding is mediated by the oligolysine terminus. Furthermore, several well-known proteins were tested for unspecific binding to the peptide modified chip surface (Fig 4B). All proteins were injected at the same mass concentration. Only for core-streptavidin and lysozyme minor binding to PeB was found showing that surface inertness is not impaired.

**Fig 5:**
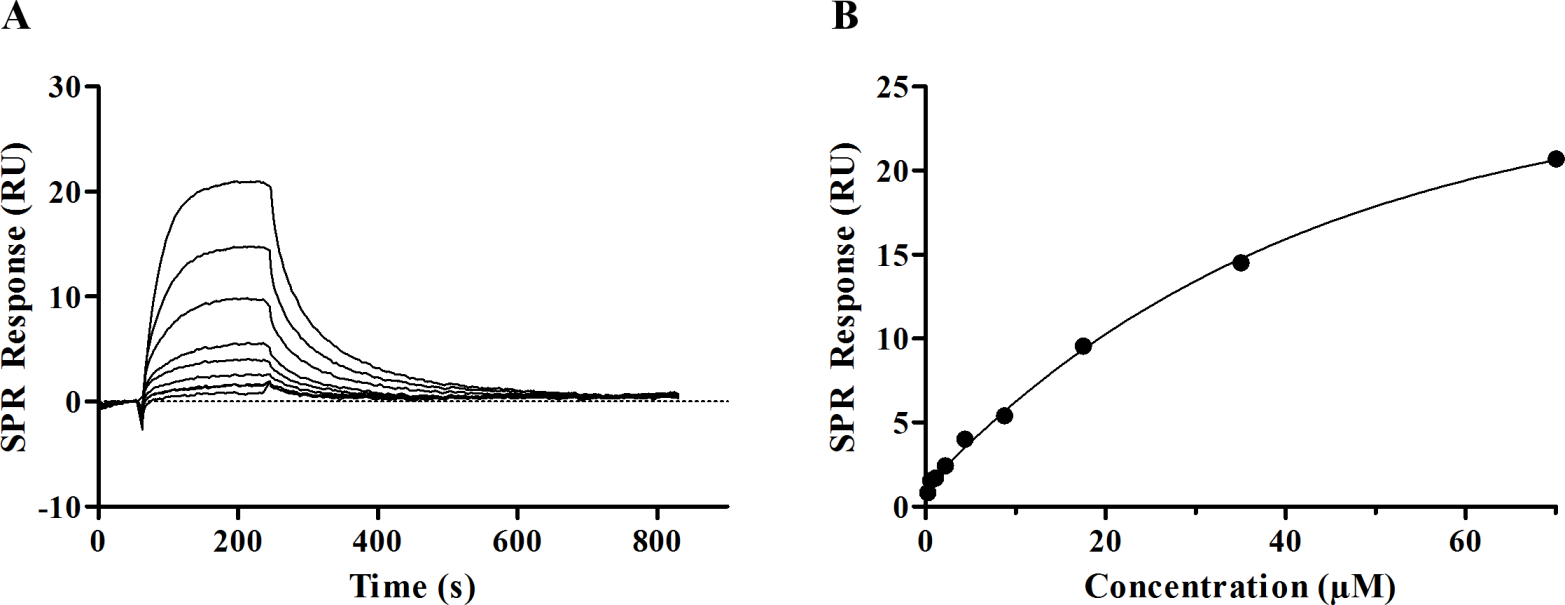
Binding of PeB to immobilized HA from Aichi H3N2. A) SPR sensorgrams of PeB binding to Aichi H3N2 HA. Peptide injections range from 0.27–70 μM. B) SPR response at equilibrium plotted against injected peptide. Data points were fitted by a steady state affinity fit curve.

Very likely, viruses bind to the peptide-covered surfaces in a multivalent manner (see Discussion). As it is not possible to calculate reliable binding constants from the multivalent binding, the inverse experiment using surface immobilized HA from Aichi H3N2 was performed. From steady-state affinity calculation for PeB a binding constant *K*_D_ of 56.8 μM (*R*_*max*_ = 35.6 RU; *chi*^*2*^ = 0.088), was obtained (Fig 5A and 5B). To assess whether PeB binds to and thus blocks the sialic binding pocket of Aichi H3N2 neuraminidase, an enzyme activity assay with the substrate MUNANA was performed (for details see Material and Methods). While the neuraminidase inhibitor DANA (N-Acetyl-2,3-dehydro-2-deoxyneuraminic acid), serving as a positive control, was able to inhibit neuraminidase activity (IC_50_ 2.2 ± 0.3 μM), PeB did not reduce the catalytic activity of the enzyme up to 360 μM peptide concentrations (S2 Fig). Thus, PeB is not compromising the accessibility to the catalytic cleft of NA.

**Fig 6:**
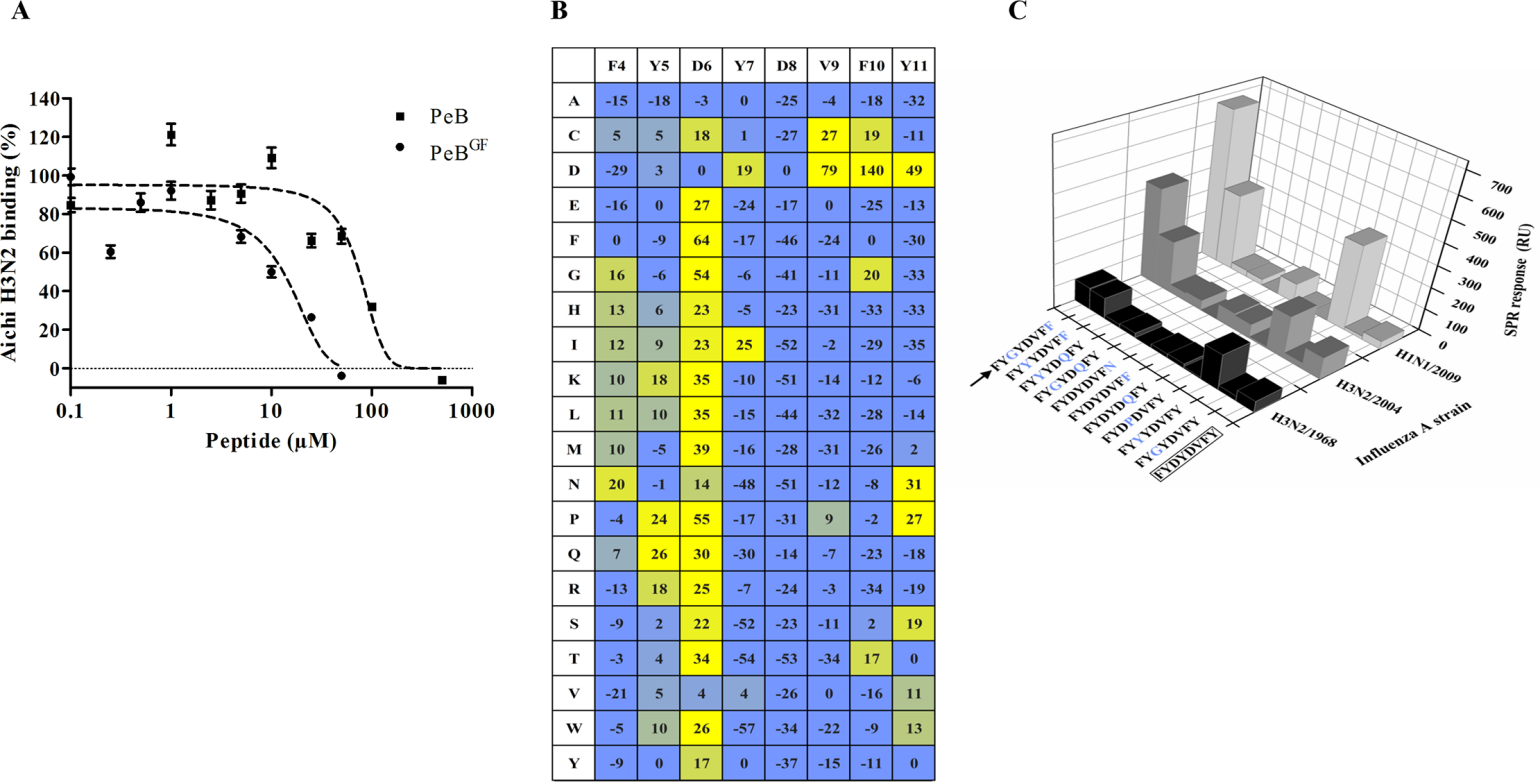
Enhancement of peptide binding affinity to HA by site-directed substitution. A) Inhibition of Aichi H3N2 binding to immobilized fetuin by PeB-Lys and PeB^GF^-Lys as determined via SPR (n = 2). Error bars indicate the SEM. Dashed lines represent a four parameter logistic fit. B) Microarray based substitutional analysis of PeB using fluorescently labeled Aichi H3N2 viruses as analytes (for further virus strains see S7 Fig). Amino acids in the first row represent that of PeB, while amino acids in the first column show the substitution. The values represent the contrast relative to fetuin as positive control (see Material and Methods). C) Binding of different viruses to selected immobilized single and double amino acid substituted variants of peptide PeB-Lys. The binding response from SPR measurements is shown (as in Fig 3).

To further support specificity of binding, inhibition of binding of Aichi H3N2 to surface immobilized fetuin by PeB was studied. Fetuin contains α-linked 2,3’- and 2,6’-sialic acids, which are the HA binding constituents on the surface of epithelial cells. As shown in Fig 6A, Aichi H3N2 binding to fetuin was inhibited by PeB. From the sigmoidal fit an *IC*_50_ value of 75 ± 13μM for PeB was calculated. Furthermore, PeB also prevented binding of Aichi H3N2 to 2,6’-sialyllactose immobilized on a SPR chip at lower concentrations than soluble 2,6‘-sialyllactose, with *IC*_*50*_ values of 120±7 μM and 1790±90 μM, respectively (S6 Fig). These results provide evidence that PeB competes successfully for the SA binding site and, thus, may serve as a virus binding inhibitor.

### Improvement of peptides by site-directed substitution

Our MD simulations have shown that the main contributions of PeB binding to HA originate from residues 4 to 12 of the peptide. In search for peptides with higher binding affinity, a full substitutional analysis using microarrays was performed. Thus, 152 PeB mutant variants were generated. In each of them, the full-length sequence was preserved except for only one amino acid from the eight loop-forming amino acids of the original PeB peptide (ARDFYDYDVFYYAMD) which was substituted with the 19 remaining natural amino acids (Fig 6B). To assess binding, Aichi H3N2 was labeled with a protein reacting fluorophore.

Most notably, substitution of D6 generally leads to an increased binding signal. This is in agreement with our MD simulations, predicting that this residue causes repulsive forces (+3.7 kcal mol^-1^) by interacting with HA. Furthermore, most substitutions (out of D, G, T) of F10, which was predicted to provide high attractive contribution (-3.0 kcal mol^-1^), reduced the obtained fluorescence signal. Although the results are essentially consistent with the theoretical predictions, we noticed a deviation: following the microarray results, aspartic acid D8 seems to be essential for binding while the MD simulation assigned it to be repulsive. Notably, for some substitutions of Y11 we observed also an enhanced binding of virus.

The microarray approach was applied to other influenza virus strains which are of higher medical relevance (S7 Fig): New York H3N2, Victoria H3N2 and California H1N1. Similar as for Aichi H3N2, most substitutions of D6 increased binding of California H1N1 and–although rather marginal—of New York H3N2. However, for the latter as well as for Victoria H3N2 substitutions of other residues caused significantly enhanced binding. For both strains all substitutions of Y11 lead to enhanced affinity.

Virus binding of selected peptide variants was evaluated by SPR to identify variants with improved binding. Particularly, we were interested in variants showing binding to various influenza virus strains. To this end, several doubly substituted variants were probed. Based on the results for monosubstituted variants (see above), we have chosen D6 and Y11 for substitution. Fig 6C shows the results for the binding of three virus strains to PeB-Lys and 10 mono- and double-substituted variants.

The best variant was PeB^GF^ with the sequence ARDFYGYDVFFYAMD. The repulsive amino acid D6 (+0.8 kcal mol^-1^) and the only slightly attractive residue Y11 (-0.1 kcal mol^-1^) (see MD simulation above) were replaced by G and F, respectively. Contribution to the total binding free energy change of every amino acid of PeB^GF^ binding to HA of Aichi H3N2 obtained by MD-simulations is shown in Fig 2B. D6G and Y11F mutations decreased the contribution of free energy changes of these residues (S4 Fig and Discussion). Y11F alters only moderately its interaction with HA, but it induces a favorable contribution change in the interaction of its neighbor residue phenylalanine 10 with HA. The side chain of residue 11 is oriented towards the solvent in PeB, due to its polar property. On the other hand, the absence of a -OH group in the residue side chain in PeB^GF^ induces a slight change in the orientation of the loop, tilting now the phenylalanine side chain towards a hydrophobic region of the HA α-helix, located between residues 160–163.

The amount of hemagglutinin bound to PeB^GF^ compared to PeB was 2-, 4- and 20-fold for Aichi H3N2, New York H3N2 and California H1N1, respectively. Specific binding of PeB^GF^ to the receptor binding site of HA was evidenced by SPR (Fig 6A, Table 2). Compared to PeB, PeB^GF^ showed a 6-fold lower *IC*_50_ (13 μM vs 75 μM) for inhibition of Aichi H3N2 binding to a fetuin-immobilized SPR surface. For PeB we observed a minor tendency to bind to immobilized fetuin, which becomes obvious from calculated inhibition values higher than 100%.

**Table 2:** Mean values of *IC*_50_ [μM] from inhibition experiments using SPR (left column), together with its SEM (n≥2). SPR binding of virus Aichi H3N2 to fetuin is shown in Fig 6A. Inhibitor constant *Ki*(HAI) [μM] from hemagglutination inhibition experiments with its SEM (n≥3). Data is plotted in Fig 7. Mean values of *IC*_*50*_ [μM] from infection inhibition (Fig 7B and 7C) and microneutralization assays (Fig 7D and 7E) are presented together with its SEM (n≥3).

Influenza strain	Inhibition (SPR)		Hemagglutination inhibition (HAI)		Infection inhibition		Neutralization	
	PeB	PeB^GF^	PeB	PeB^GF^	PeB	PeB^GF^	PeB	PeB^GF^
Aichi H3N2	75 ± 13	13 ± 3	235 ± 15	32 ± 1	32 ± 5	25 ± 6	6 ± 1	7 ± 1
Rostock H7N1	40 ± 10	30 ± 5	63 ± 0	23 ± 6	216 ± 11	94 ± 6	177 ± 6	96 ± 14

### Peptide mediated inhibition of virus infection

**Fig 7:**
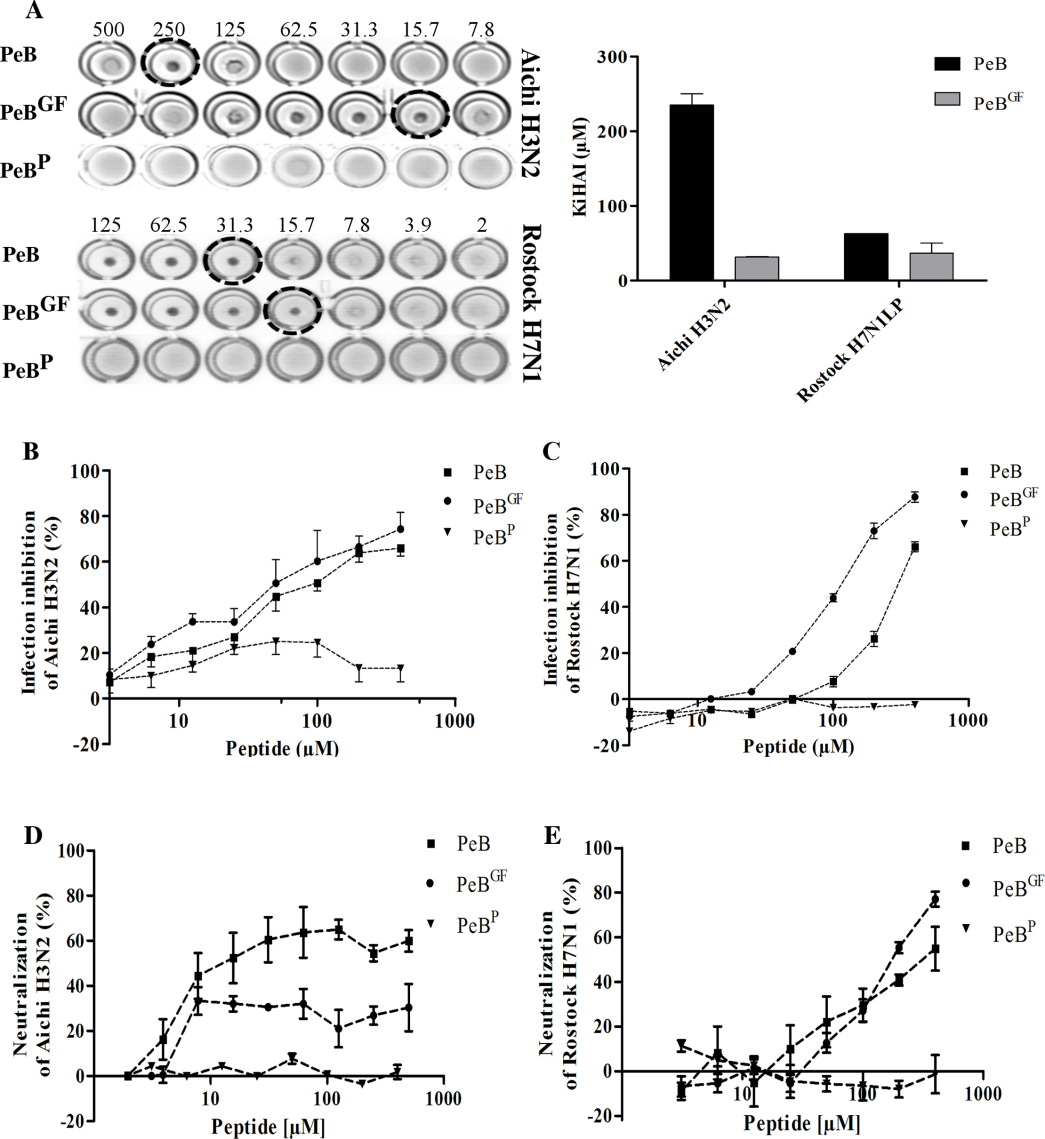
Peptide mediated inhibition of virus binding and infection. A) Inhibition of Aichi H3N2 and Rostock H7N1 mediated hemagglutination by PeB and PeB^GF^. *K*_i_(HAI) values represent the minimum concentration for full hemagglutination inhibition. B and C) Infection inhibition of MDCK II cells with PeB, PeB^GF^ and PeB^P^ (MOI 0.05) with Aichi H3N2 (B) or Rostock H7N1 (C). Infection was followed by measuring the decrease of absorption of the MTS reagent. Error bars represent the SEM with n≥3. D and E) Neutralization of Aichi H3N2 (D) and Rostock H7N1 (E) by PeB and PeB^GF^, while PeB^P^ served as a control. Error bars represent the SEM with n = 3.

To characterize the potential of antibody derived peptides to prevent virus replication, we studied virus binding and infection of host cells in the presence of PeB, PeB^GF^ and PeB^P^. The latter is assumed to be a structurally distinct variant due to the presence of a proline residue, used as a negative control. Both PeB and PeB^GF^ efficiently prevented Aichi H3N2 mediated aggregation of red blood cells (RBC) as assessed by HAI (Fig 7A). However, PeB^GF^ was much more efficient in comparison to PeB. The inhibitory constant *K*_*i*_(HAI) was 235 μM and 31.9 μM for PeB and PeB^GF^, respectively. Furthermore, we could show binding inhibition of Rostock H7N1 virus to turkey erythrocytes at *Ki*(HAI) values of 47 μM for PeB and 23 μM for PeB^GF^ (Table 2). Hemagglutination by both viral strains was not inhibited by PeB^P^.

Both peptides also protected efficiently MDCK II cell from infection (MOI 0.05) by Aichi H3N2 (Fig 7B) and Rostock H7N1 (Fig 7C). PeB^P^ did not compromise the infection at all. Importantly, all three peptides did not impair cell viability within 24 h treatment with peptides up to the maximum concentration (400 μM) studied (S8 Fig).

Similar results were obtained from a microneutralization assay, in which the peptide inhibitors are present in solution throughout the whole 24 h incubation (Fig 7D and 7E and Table 2). Here, viral nucleoprotein expression was detected by immunostaining of infected cells, and the results were compared to untreated, infected cells. The IC_50_ values of Rostock H7N1 were similar to that obtained by the MTS based infection inhibition assay. However, the IC_50_ values for Aichi H3N2 were somewhat lower (3–5 fold) with respect to the MTS assay.

Although micromolar *IC*_*50*_ values seem to be rather high concentrations from the point of view of medical applications, the results are very promising for developing multivalent inhibitors (see Discussion).

**Fig 8:**
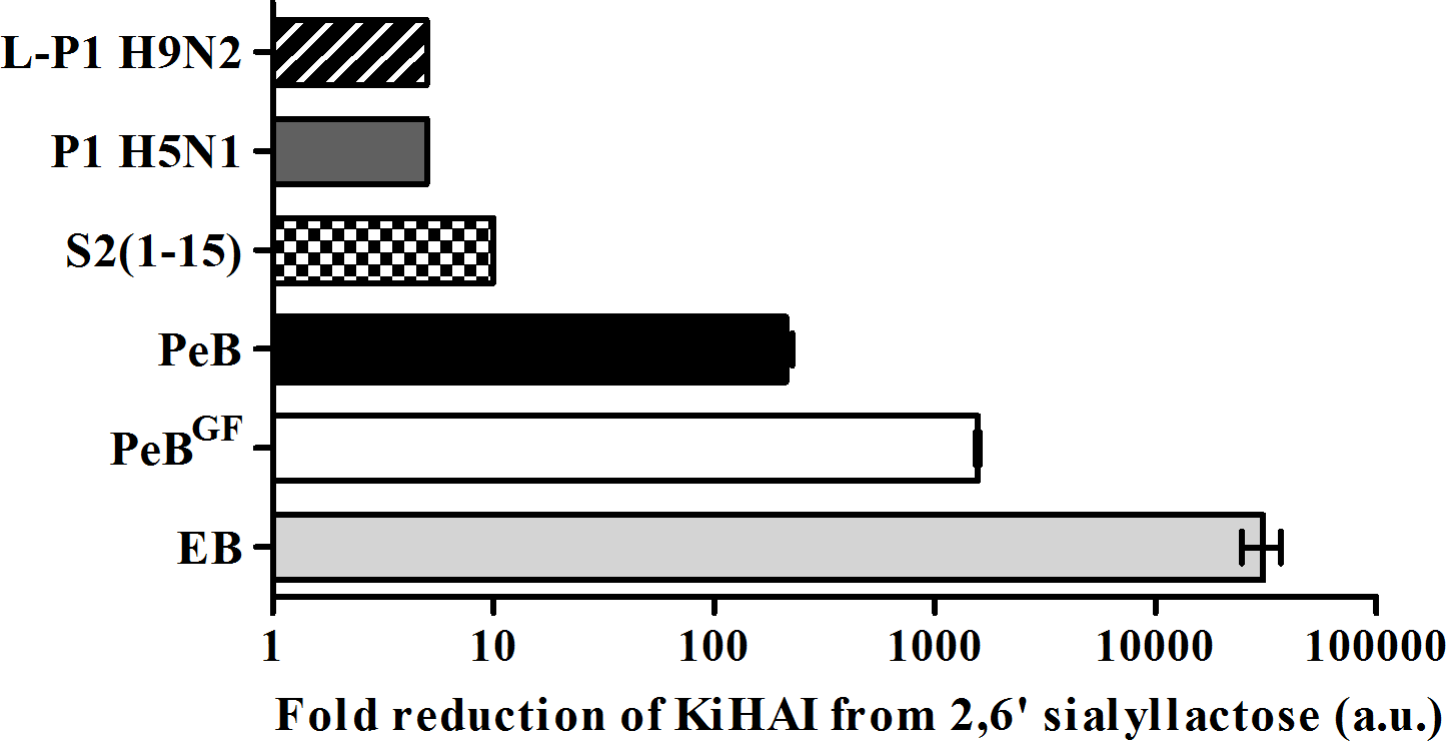
Inhibitory potency of PeB and PeB^GF^ as compared with known anti-influenza peptides. Inhibition of hemagglutination based on their *K*_i_(HAI) of different peptides as compared to 2,6‘-sialyllactose (EB: RRKKAAVALLPAVLLALLAP [45], s2(1–5): ARLPRTMVHPKPAQP [17], Phage 1 (P1) H5N1: HAWDPIPARDPF [18], Phage 1 (L-P1) H9N2: NDFRSKT [16]. Error bars represent the SEM (n = 3).

To evaluate the inhibitory potential of the antibody derived peptides in relation to that of already published HA binding peptides, we compared these peptides in the HAI assay (Fig 8) [16–18, 20, 45]. We normalized *K*_i_(HAI) values to that of 2,6-sialyllactose as the natural receptor for H3. With reduction of *K*_i_(HAI) by 200 and 1600 fold, PeB respectively PeB^GF^ showed superior potential compared to other published peptides, with the exception of the “entry blocker” EB. As in previous experiments, peptide PeB^GF^ showed an higher inhibitory potency than PeB as observed by its 8-fold lower *K*_i_(HAI). Additionally both peptides showed lower *K*_i_(HAI)-values than peptides obtained by phage display against H3N2 (s2(1–5)), H5N1 and H9N2. Only the entry blocker EB showed superior hemagglutination inhibition, revealed by its 20-fold lower *K*_i_(HAI) as compared to PeB^GF^_._ However, due to its amphiphilic character, EB forms micelles and thus may act in a multivalent manner [45]. Therefore, it inhibits Aichi H3N2 hemagglutination more efficiently than other monovalent peptides.

For the sake of completeness we mention that Mucroporin M-1 showed strong hemolytic effects and strong interference with gold surfaces, which made it impossible to obtain reliable data [20].

## Discussion

### Selection of antibody-derived peptides

Our aim was to obtain peptide inhibitors that recognize the conserved region of the sialic acid binding pocket of HA with a broader specificity to cover several influenza virus strains. The three CDRs of the VH-chain of antibody HC19 against hemagglutinin of influenza virus Aichi H3N2 were used as templates to design peptides being a potential inhibitor of virus binding to host cells. We used complementary experimental approaches as SPR and peptide microarrays, and MD to select appropriate peptides and to enhance their affinity to HA by site directed substitutions of amino acids. This was validated by assessing their potential to prevent virus binding to cell surfaces and, eventually, infection of host cells.

Three peptides–PeA, PeB and PeC–have been derived from the three CDR sequences of the HC19 antibody’s heavy chain. In agreement with predictions from our MD calculations of peptide-HA complexes, we found by SPR experiments that binding of surface immobilized peptide PeB to HA from Aichi H3N2 was superior to PeC (Fig 3). Therefore, and as PeB matches the conserved sialic acid binding site better than PeC (PDB 2vir) [46], subsequent studies were performed with PeB and its derivatives. Inhibition of virus binding to surface immobilized fetuin and 2,6’-sialyllactose by PeB confirmed the specific binding of the peptides to the sialic acid binding pocket of HA. The *IC*_50_ values are in the range of 75–120 μM, depending on the assay, which is about one order of magnitude lower than the *IC*_50_ of α-2,6-sialyllactose.

An important observation which may be also of interest for upcoming studies is that we found specific binding of viruses to surface immobilized peptides consisting only of amino acids representing the PeB core sequence, and a four lysine long linker (Fig 3). Nevertheless, PeB and its derivatives harboring only those amino acids which are essential for the recognition of SA are presumably not efficient binders in their monomeric form. The affinities of the peptides are in the micromolar range, whereas the reported *K*_*D*_ value of antibody HC19 binding to hemagglutinin from Aichi H3N2 is 28 ± 8 nM [47]. However, weak binding interactions for such shorter monomeric peptides could be enhanced by a multivalent interaction as it occurs very likely in the SPR based experiments, where binding of even 6.5∙10^8^ viruses ml^-1^ to immobilized PeB could be detected (Fig 3). Extensions of the C- and N-termini of the CDR sequences could be important for (i) the formation of a stable peptide structure, which exposes the CDR loop region in its native arrangement or (ii) direct involvement in binding and foster interaction with HA.

### Substitutional analysis and improvement of binding affinity of PeB

According to the MD simulations PeB adopts a strand-loop-strand conformation with the loop consisting of residues D6 to Y11. As shown in Fig 2B, residues within and adjacent to the loop (Y5 to Y12) include the residues yielding the largest contributions to the binding free energy. Most of these contributions are attractive (negative free energies changes) but some are also repulsive (positive free energies changes). The contributions of each amino acid to the free energy calculated by MM-PBSA were largely consistent with the results of microarray-based substitutional analysis, despite the described discrepancy for D8. Possibly, substitution of D8 changes the secondary structure leading to a lower affinity.

In the light of improving the affinity of PeB and to enable a broader specificity we also probed the binding of the peptide to HA of other virus strains by microarray-based substitutional analysis. We identified that Y11 is also an important residue for improving binding. Based on that, we investigated the affinity of selected variants of PeB having two substitutions. In this screen, we identified PeB^GF^ as the best binder. The two substitutions, D6G and Y11F, altered the ligand moiety and its binding contacts, resulting in favorable changes to the contribution to the free energy change of residues 6, 8 and 10 (Fig 2B). The mutation of residue 6 (D6G), replacing a charged amino acid by a neutral one, resulted in two major changes. Firstly, it induces a decrease of its own electrostatic contribution to the binding free energy, but such a change is largely overcome by an increase in the contribution to the polar solvation free energy change (S3 Fig). On the other hand, one should notice that residues 6 and 8 are located in close contact to each other in the loop region. As a consequence for PeB, this involves repulsive interaction between two negatively charged residues (D6 and D8). The replacement of D6 by an uncharged residue in PeB^GF^ probably induces a slight reorganization in the loop structure, favoring van der Waals and polar solvation interaction contributions between this residue and the HA moieties (S3 Fig).

### Inhibition of virus binding and infection by peptides

Due to its orientation towards the SA binding region of HA, CDR3 sequences of antibody HC19 [26, 27] against HA of Aichi H3N2 are promising candidates for getting variants with broad influenza strain specificities. Indeed, both peptides PeB and PeB^GF^ were found to bind to HA of other strains (Rostock H7N1, New York H3N2, and California H1N1). It is worth to note that all HAs of different influenza subtypes, which could be bound and inhibited by peptides PeB or PeB^GF^ reveal strong structural similarities, especially for those amino acids involved in peptide binding (S2 Table). This close relation supports the wide binding ability and inhibitory potency of PeB for a variety of influenza A strains.

Such broader specificity is also supported by the similarity between the VH-CDR3 of the mouse antibodies against Aichi H3N2 hemagglutinin in PDB 1ken (antibody HC63, amino acids 97–109: AAFYYDYDFFFDY) and PDB 2vir, antibody HC19, amino acids 97–109: RDFYDYDVFYYAM), and also a human antibody against H3: (EGDYDILTGYYYYFDY), respectively [26, 48, 49]. Notably, there is enrichment of aromatic amino acids Tyr and Phe, and of Asp. In line with these findings, a recently published human antibody against H1 shows a higher proportion of aromatic amino acids in the HCDR3 domain, too [50]. The crucial role of an Asp residue and hydrophobic amino acids in the HA binding of many receptor mimicking antibodies is highlighted by Lee et al [13].

As shown exemplarily for Aichi H3N2 and Rostock H7N1 inhibition of virus binding by peptides leads to a strong protection from virus infection of host cells. In the MTS based infection inhibition assay PeB^GF^ was found to be superior with respect to PeB, too. A similar observation was made for Rostock H7N1 when using the microneutralization assay, which provides a direct measure for viral replication. However, for Aichi H3N2 we did not find a preference for PeB^GF^ indicating strain dependent differences of the antiviral potential of the peptides.

The inhibitory potential of our antibody derived peptides demonstrated a remarkable performance compared to other published peptides binding to HA (Fig 8). As summarized in Table 2, PeB and PeB^GF^ provides better inhibition against Aichi H3N2 compared to Rostock H7N1, with the exception of the results from the HAI assay. However, we should take into consideration that the performance of a peptide as inhibitor depends on many factors. As a consequence, the hemagglutination inhibition efficiency may not necessarily be stronger against Aichi H3N2, even though the peptide was derived from an antibody binding to this influenza strain. One can speculate that Rostock H7N1 is more prone to neutralization by blocking this binding site, while for Aichi H3N2 PeB and PeB^GF^ can be displaced more easily in a binding competition experiment with erythrocytes. This trend could also be explained by the use of erythrocytes derived from different species (see Material and Methods). Similar strain-dependent efficiencies have been obtained by López-Martinez et al. [22]. Although a peptide was designed against broadly conserved epitopes, the antiviral efficiency was not the same against all tested serotypes (three different H1N1 strains and H5N2).

Short oligomeric biomolecules with sufficient affinity are of high interest for diagnostics and drug design, as development, synthesis and further chemical modifications are easy to implement.

Linear short CDR derived peptides as used here can be expected to differ from the loop structure they adopt within the antibody, leading to lower affinity. However, there are promising ways to largely improve them. Cyclisation of CDR peptides using a D-Pro-L-Pro template has been shown to force them into canonical conformations [51]. In this report, the L3 loop of the HC19 antibody adopted a hairpin structure and an aromatic T-stacking as in the antibody crystal structure. This and other strategies to generate peptidomimetics by stabilizing or mimicking turns, ß-sheets and helices, have been recently reviewed [52].

Whereas the gain in affinity by cyclization of a peptide is limited by the affinity of the native structure, larger improvements can be achieved by multivalency, especially when a virus with a high target protein density on its surface has to be bound [53–56]. For example, sialyl-containing oligosaccharides constituting the natural receptors of hemagglutinin on mammalian epithelial cells, exhibit a millimolar 1:1 affinity, but due to multivalent virus-cell attachment the binding is strong enough to enable infection. Effective inhibition of influenza virus infection by multivalent sialic-acid-functionalized gold nanoparticles and other glycoarchitectures has been described [57, 58]. More recently, a trivalent glycopeptide mimetic containing three sialyl residues matching the three sialyl binding sites in a HA trimer, was reported to bind to H5 hemagglutinin 4000 fold better than the monomer [59]. Recently, we demonstrated that the antiviral effectivity of acylated PeB^GF^ can be increased, upon presentation in a multivalent fashion by 10-fold against Rostock H7N1 and by 20-fold against Aichi H3N2 [60]. Further, multivalent presentation of the short s2 peptide (ARLPR) with a dendrimer resulted in sub-micromolar inhibitory activities [61].

## Conclusions

The antibody derived peptide PeB and its mutant variant PeB^GF^ are promising hemagglutinin binding peptides, which both could have potential for application in viral diagnostics and therapeutics. Infection inhibition could be achieved at micromolar concentrations. Strategies to improve the peptide structure and to apply the peptides for developing multivalent binders with high affinities are discussed.

Finally, the workflow presented involving complementary experimental and theoretical approaches could be a template for the development of antiviral agents, which could be also applied for influenza detecting biochips. In a coevolutionary approach the peptide’s primary sequence may also be adapted to seasonal upcoming mutations of the influenza virus with minor additional efforts, costs and in short time without the additional development of novel monoclonal antibodies.

## Supporting Information

S1 Fig Analysis of peptide (PeB) structure. Solutions of PeB have been analyzed for structural features using circular dichroism (CD) spectroscopy (A) at physiological pH (7.4) and at pH 2, below the isoelectric point of the peptide. Further, the melting temperature profile of peptide PeB under both pH conditions was measured (B).
(TIF)

S2 Fig Neuraminidase activity assay. Enzyme activity in the presence of the inhibitor DANA (N-Acetyl-2,3-dehydro-2-deoxyneuraminic acid) and PeB. Relative fluorescence of 4-Methylumbelliferone was measured after enzymatic conversion of MUNANA (4-Methylumbelliferyl-N-acetyl-α-D-neuraminic acid) by neuraminidase following a known protocol [30]. Data show mean values of triplicate experiments with SEM (n ≥ 3), dashed line represents a sigmoidal fit.
(TIF)

S3 Fig Per-residue decomposition of binding free energy. Contributions of individual residues of PeB (black) and PeB^GF^ (grey) to the binding free energies of the corresponding PeB-HA or PeB^GF^-HA complexes. A) Electrostatic, B) van der Waals and C) polar contributions to the total binding free energy change. The mutation of residue 6 (D6G) replacing a charged amino acid by a neutral one, decreases the electrostatic contribution, but such a change is largely overcome by an increase in the contribution to the solvation free energy change, as calculated. Mutation of amino acid 11 (Y11F) appears not to alter directly the contribution of the interaction between this residue and the HA, but it induces a favorable free energy change contribution in the interaction between residue 10 and the HA, essentially by a more favorable van der Waals interaction energy change between uncharged residues.
(TIF)

S4 Fig Contributions of individual residues of HA to the binding free energies of the corresponding PeB-HA complexes. A) Wildtype HA (Aichi H3N2), and two single mutants B) T155I and C) T131I.
(TIF)

S5 Fig SPR sensorgrams for Aichi H3N2 binding to peptides. Immobilized peptides were A) PeA-Lys (0.77 pmol mm^-2^), B) PeB-Lys (0.57 pmol mm^-2^) and C) PeC-Lys (1.93 pmol mm^-2^). Injection of virus was initiated at t = 69 s and terminated at t = 569 s. Numbers indicate virus injections at concentrations of up to 50 μg ml^-1^.
(TIF)

S6 Fig Inhibition of binding of Aichi H3N2 viruses to α-2,6’-sialyllactose. Biotinylated α-2,6’-sialyllactose was immobilized on a neutravidin modified surface (650 RU). Virus was preincubated with peptide PeB or α-2,6’-sialyllactose before injection. Lines represent a sigmoidal fit model of the data (mean values of duplicate experiments).
(TIF)

S7 Fig Substitutional analysis of peptide PeB using additional influenza strains. Labeled influenza California H1N1 (left), New York H3N2 (middle) and Victoria H3N2 (right) were used as analytes. Numbers represent mean value of the contrast relative to contrast for positive control fetuin. False-colors are used to illuminate fluorescence intensities, color changes from blue (lower) to yellow (higher intensity than PeB). Data just represent qualitative relation between PeB mutants within single influenza strains. Quantitative comparison between different strains is not valid due to the varying types of samples, while precise quantitation fails due to unknown ligand density (most likely varying per peptide).
(TIF)

S8 Fig Cell viability of MDCK II in the presence of peptides. MDCK II cells were treated 24 h with peptides PeB, PeB^GF^, and PeB^P^ before cell viability was assessed by a MTS reagent.
(TIF)

S1 Table Proteins used for peptide binding specificity. (DOCX)

S2 Table Alignment of HA sequences of used influenza viruses. Amino acids involved in binding of PeB as obtained from MD-simulations are highlighted in gray. PDB2vir: sequence obtained from protein database; A/mute/swan/R901/06-H7N1: sequence obtained from Prof. Harder (Friedrich-Loeffler-Institut, Riems, Germany); all other sequences were obtained from influenza virus resource (IVR) as indicated by their accession numbers [62]. While L219 is identical for all subtypes, S159 and E215 are replaced in some cases by functional closely related T or D, respectively. The sequence differ mostly in N160, which is replaced by S, A or V. The N160 substitutions could be the main reason for differences in the observed binding ability.
(DOCX)
