# Supplementary figures and images for: Anti-Hemagglutinin Antibody Derived Lead Peptides for Inhibitors of Influenza Virus Binding
Source: PLoS One. 2016 Jul 14;11(7):e0159074. doi: 10.1371/journal.pone.0159074 (PMC4944999; doi:10.1371/journal.pone.0159074)

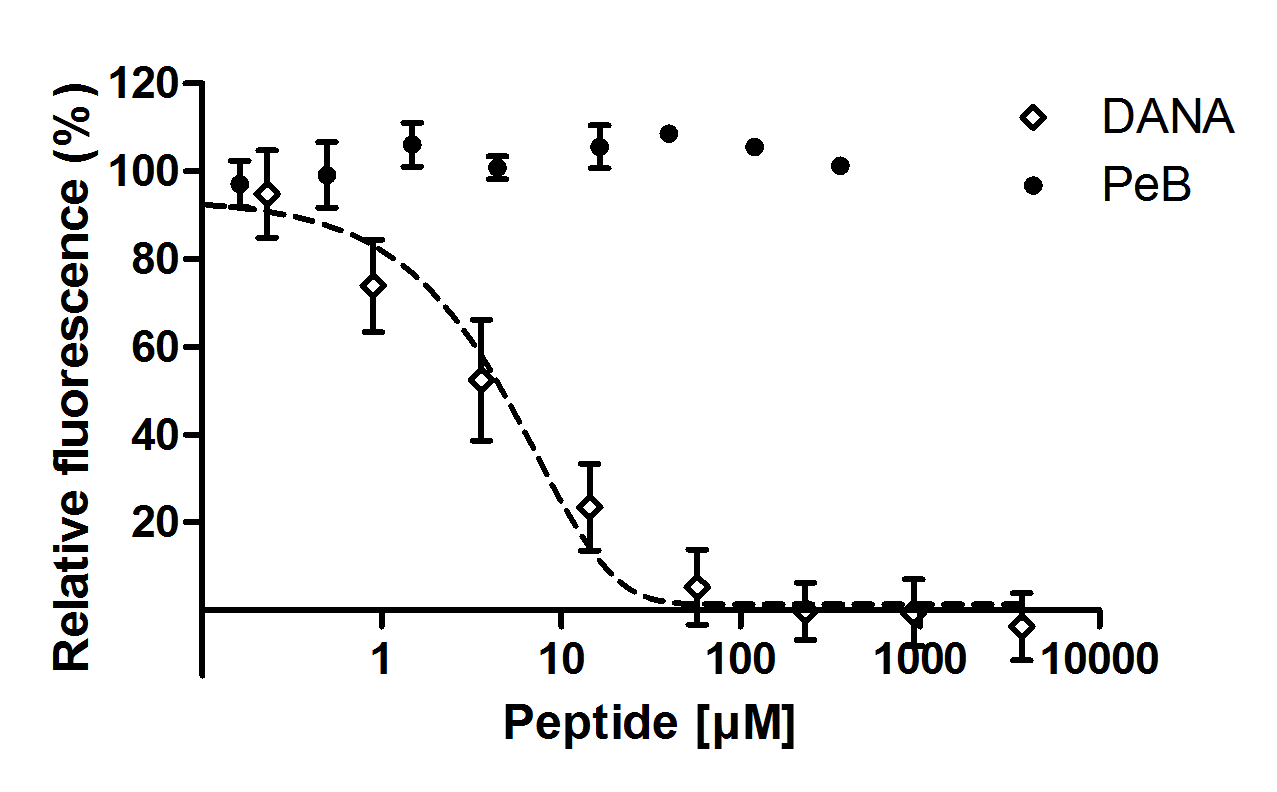

Supplement: S1 Fig — Solutions of PeB have been analyzed for structural features using circular dichroism (CD) spectroscopy (A) at physiological pH (7.4) and at pH 2, below the isoelectric point of the peptide. Further, the melting temperature profile of peptide PeB under both pH conditions was measured (B). (TIF) [file pone.0159074.s001.tif]

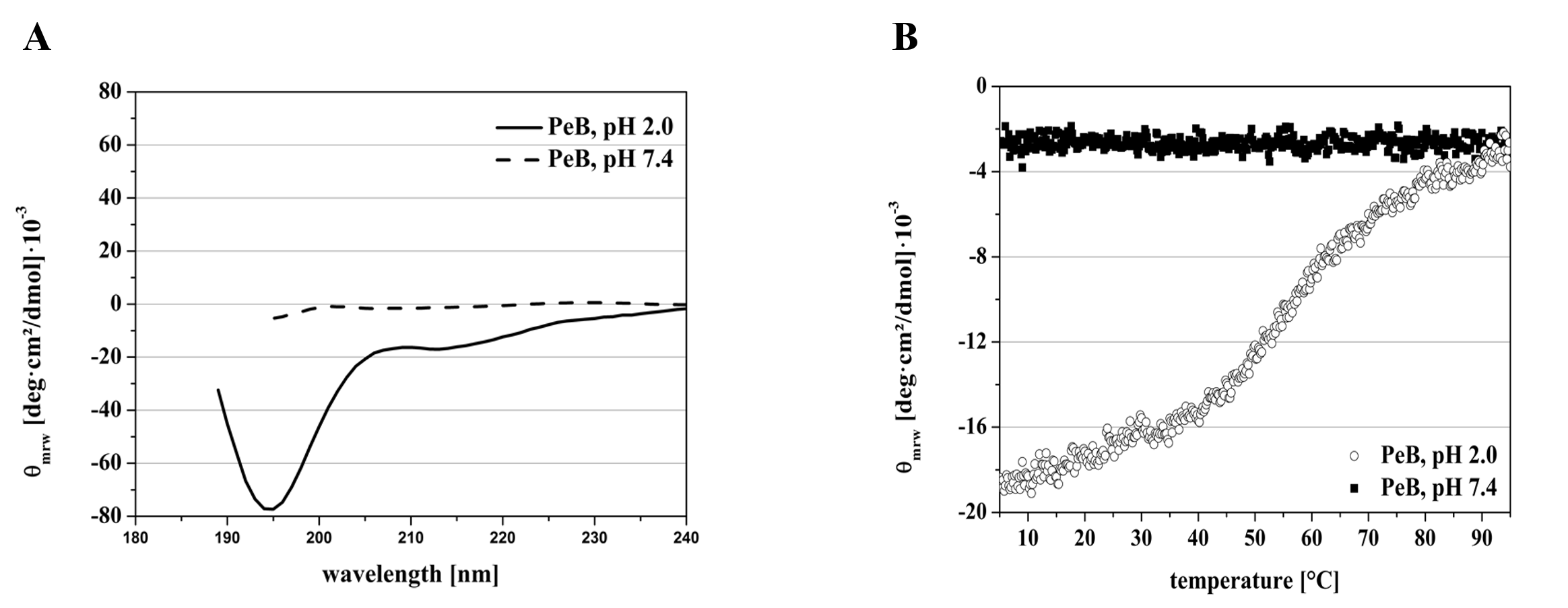

Supplement: S2 Fig — Enzyme activity in the presence of the inhibitor DANA (N-Acetyl-2,3-dehydro-2-deoxyneuraminic acid) and PeB. Relative fluorescence of 4-Methylumbelliferone was measured after enzymatic conversion of MUNANA (4-Methylumbelliferyl-N-acetyl-α-D-neuraminic acid) by neuraminidase following a known protocol [30]. Data show mean values of triplicate experiments with SEM (n ≥ 3), dashed line represents a sigmoidal fit. (TIF) [file pone.0159074.s002.tif]

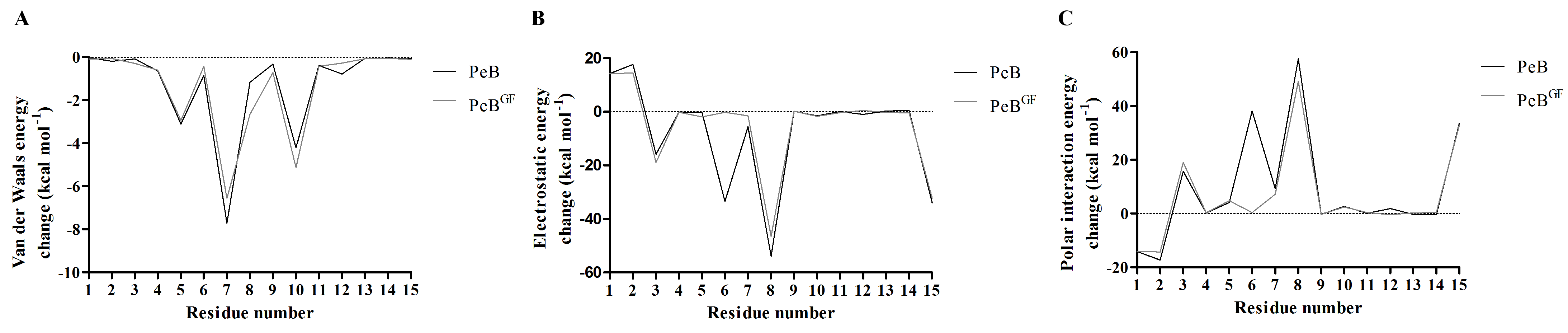

Supplement: S3 Fig — Contributions of individual residues of PeB (black) and PeBGF (grey) to the binding free energies of the corresponding PeB-HA or PeBGF-HA complexes. A) Electrostatic, B) van der Waals and C) polar contributions to the total binding free energy change. The mutation of residue 6 (D6G) replacing a charged amino acid by a neutral one, decreases the electrostatic contribution, but such a change is largely overcome by an increase in the contribution to the solvation free energy change, as calculated. Mutation of amino acid 11 (Y11F) appears not to alter directly the contribution of the interaction between this residue and the HA, but it induces a favorable free energy change contribution in the interaction between residue 10 and the HA, essentially by a more favorable van der Waals interaction energy change between uncharged residues. (TIF) [file pone.0159074.s003.tif]

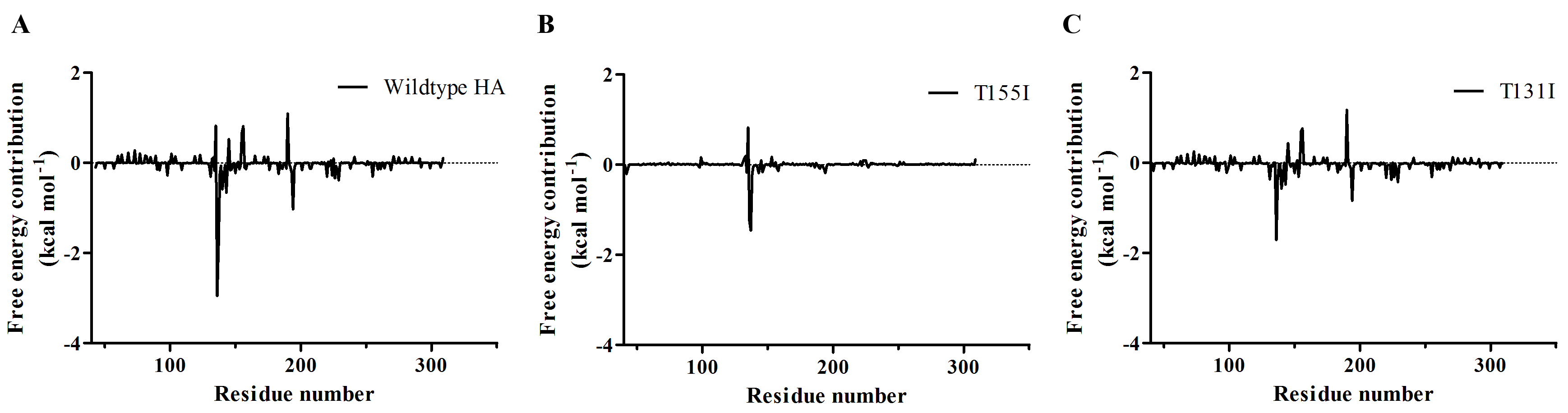

Supplement: S4 Fig — A) Wildtype HA (Aichi H3N2), and two single mutants B) T155I and C) T131I. (TIF) [file pone.0159074.s004.tif]

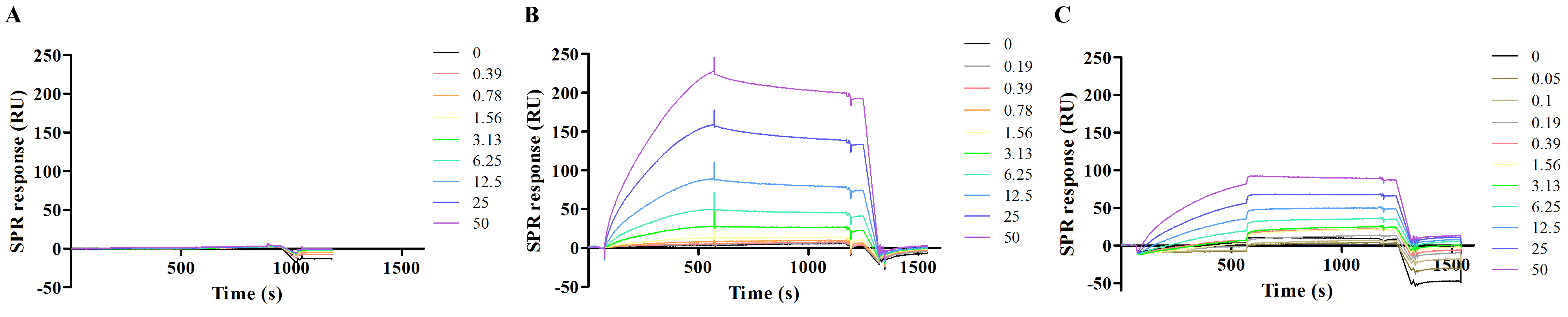

Supplement: S5 Fig — Immobilized peptides were A) PeA-Lys (0.77 pmol mm-2), B) PeB-Lys (0.57 pmol mm-2) and C) PeC-Lys (1.93 pmol mm-2). Injection of virus was initiated at t = 69 s and terminated at t = 569 s. Numbers indicate virus injections at concentrations of up to 50 μg ml-1. (TIF) [file pone.0159074.s005.tif]

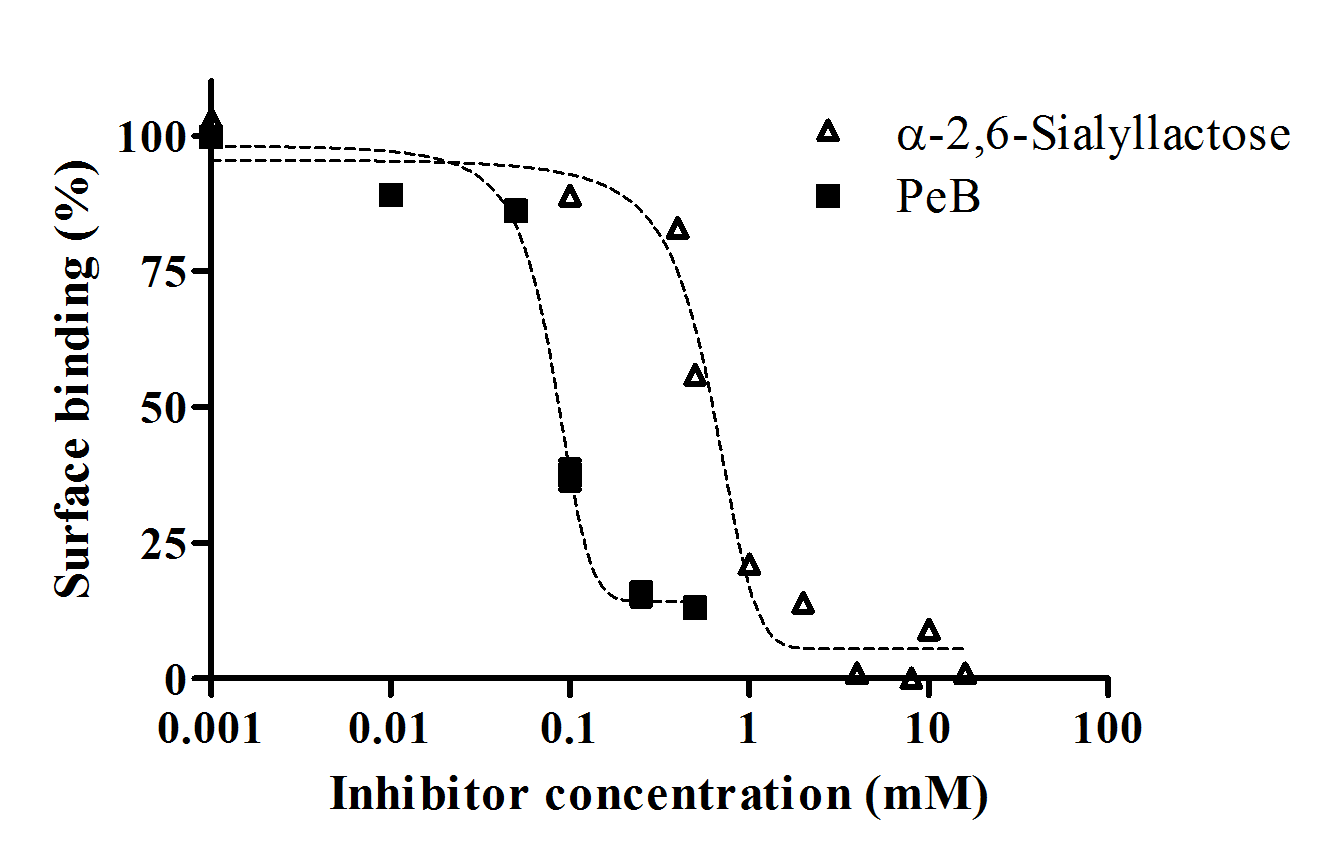

Supplement: S6 Fig — Biotinylated α-2,6’-sialyllactose was immobilized on a neutravidin modified surface (650 RU). Virus was preincubated with peptide PeB or α-2,6’-sialyllactose before injection. Lines represent a sigmoidal fit model of the data (mean values of duplicate experiments). (TIF) [file pone.0159074.s006.tif]

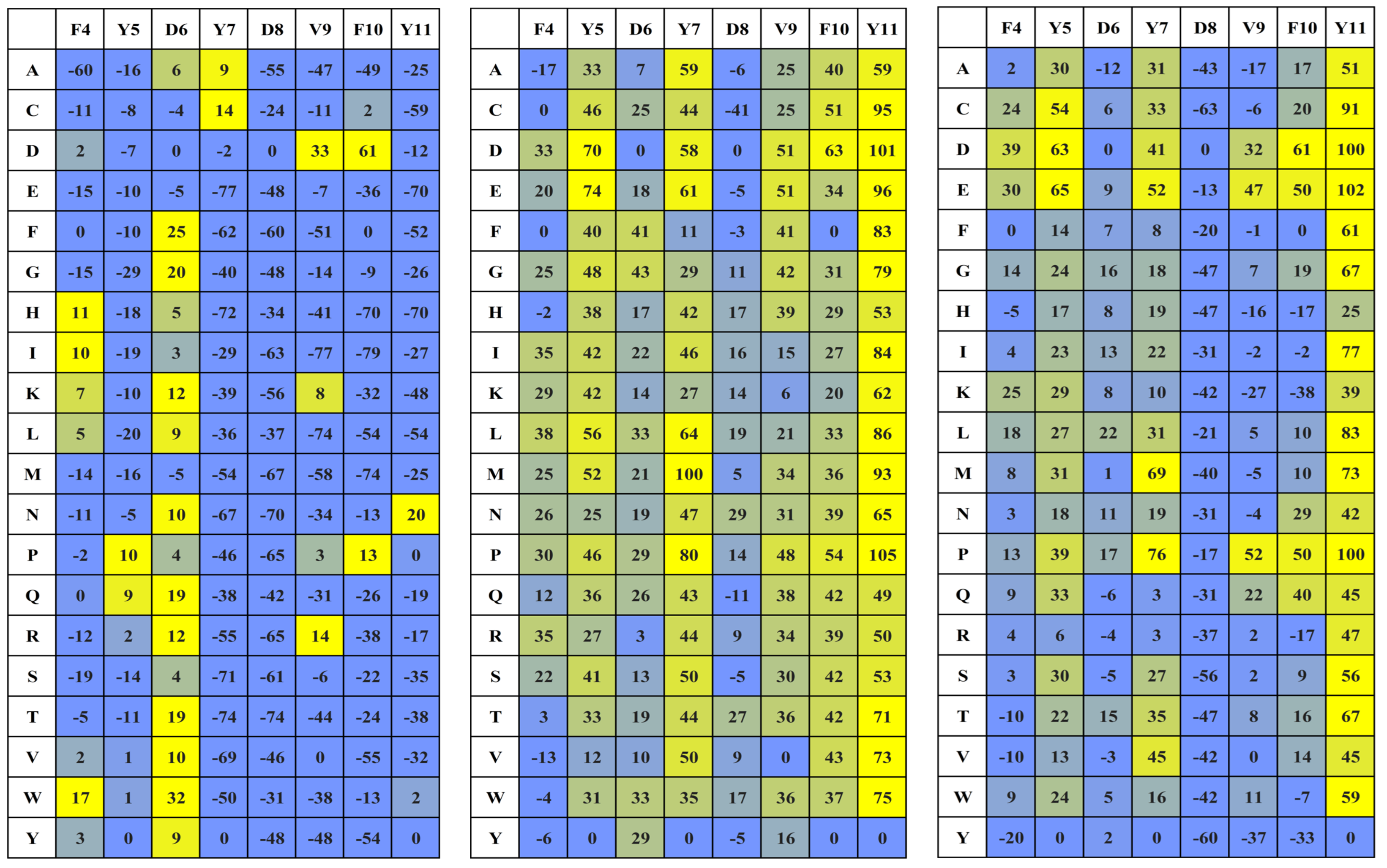

Supplement: S7 Fig — Labeled influenza California H1N1 (left), New York H3N2 (middle) and Victoria H3N2 (right) were used as analytes. Numbers represent mean value of the contrast relative to contrast for positive control fetuin. False-colors are used to illuminate fluorescence intensities, color changes from blue (lower) to yellow (higher intensity than PeB). Data just represent qualitative relation between PeB mutants within single influenza strains. Quantitative comparison between different strains is not valid due to the varying types of samples, while precise quantitation fails due to unknown ligand density (most likely varying per peptide). (TIF) [file pone.0159074.s007.tif]

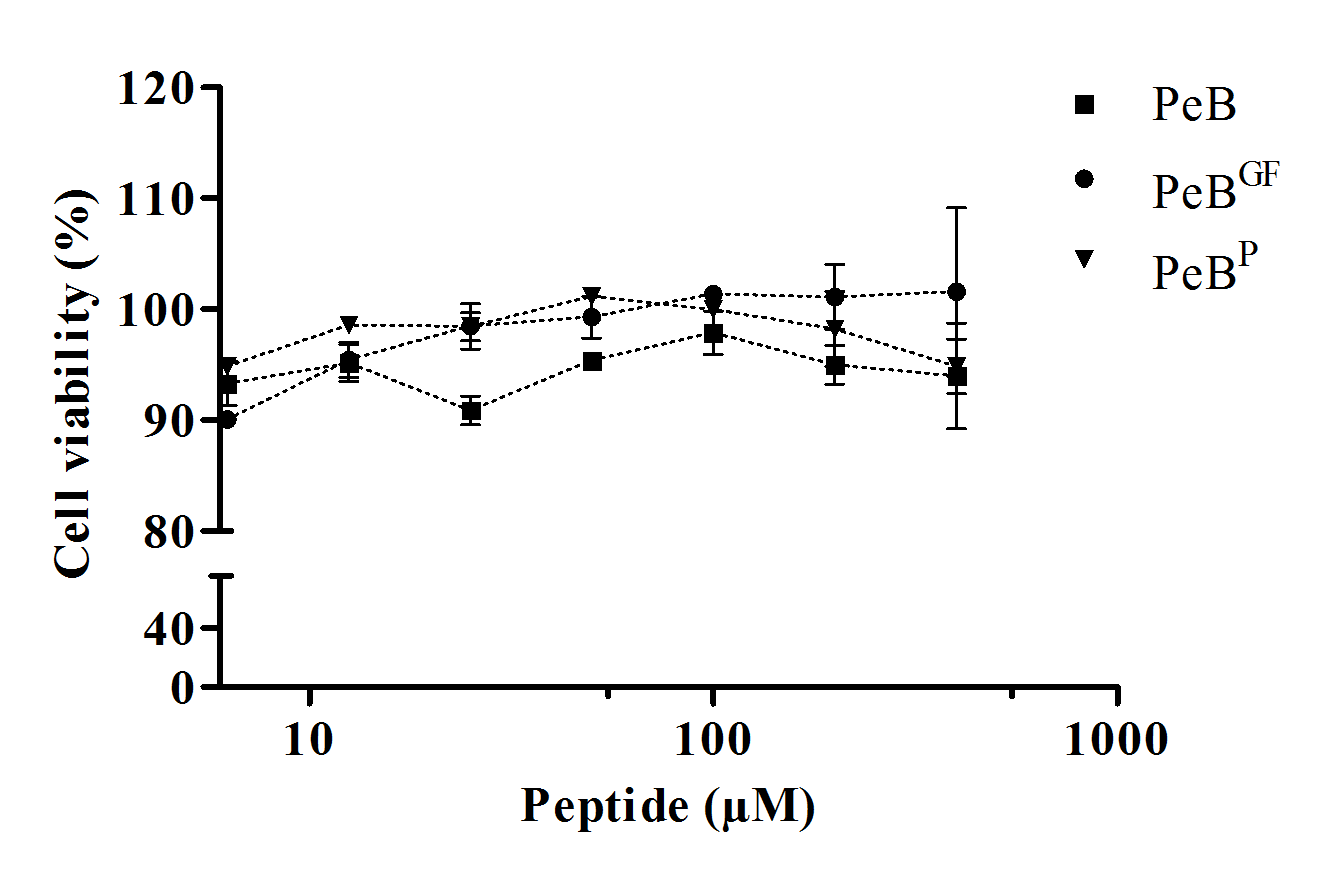

Supplement: S8 Fig — MDCK II cells were treated 24 h with peptides PeB, PeBGF, and PeBP before cell viability was assessed by a MTS reagent. (TIF) [file pone.0159074.s008.tif]
