# Supplementary material for: Anti-Hemagglutinin Antibody Derived Lead Peptides for Inhibitors of Influenza Virus Binding
Source: PLoS One. 2016 Jul 14;11(7):e0159074. doi: 10.1371/journal.pone.0159074 (PMC4944999; doi:10.1371/journal.pone.0159074)
Supplement: S1 Table — (DOCX) [file pone.0159074.s009.docx]

**S1 Table. Proteins used for peptide binding specificity.**

| **Protein** | **Source** | **Supplier, Order No.** | **Mol. Mass (kDa)** | **Isoelectric Point** |
| --- | --- | --- | --- | --- |
| Alcohol dehydrogenase | yeast | Sigma-Aldrich, A3263 | 141-152 | 5.4-5.8 |
| Bovine serum albumin | bovine serum | Sigma-Aldrich, A1933 | 66 | 4.7 |
| Casein | bovine milk | Sigma-Aldrich, C5890 | 19-25 | 4.1-5.8 |
| Streptavidin | *S. avidinii* | Applichem, A1495 | 60 | n.a. |
| Cytochrome C | horse heart | Sigma-Aldrich C2506 | 12.4 | 10.0-10.5 |
| Human serum albumin | human serum | Sigma-Aldrich, A8763 | 66.2-66.4 | 4.7 |
| Hyaluronidase | bovine testis | Sigma-Aldrich 53720 | 60 | n.a. |
| Insulin | bovine pankreas | Sigma-Aldrich, I-5500 | 5.7 | 5.3 |
| Lysozyme | hen egg white | Sigma-Aldrich, 62970 | 14.4 | 9-11 |
| Neutravidin | hen egg white | Thermo Fisher, 31000 | 60 | 6.3 |
| Pectinase | *Rhizopus sp.* | Applichem, A1495 | n.a. | n.a. |
| Ribonuclease A | bovine pancreas | Sigma-Aldrich, R5503 | 13.7 | 9.6 |

n.a. = not available
