# Supplementary material for: Anti-Hemagglutinin Antibody Derived Lead Peptides for Inhibitors of Influenza Virus Binding
Source: PLoS One. 2016 Jul 14;11(7):e0159074. doi: 10.1371/journal.pone.0159074 (PMC4944999; doi:10.1371/journal.pone.0159074)
Supplement: S2 Table — Amino acids involved in binding of PeB as obtained from MD-simulations are highlighted in gray. PDB2vir: sequence obtained from protein database; A/mute/swan/R901/06-H7N1: sequence obtained from Prof. Harder (Friedrich-Loeffler-Institut, Riems, Germany); all other sequences were obtained from influenza virus resource (IVR) as indicated by their accession numbers [62]. While L219 is identical for all subtypes, S159 and E215 are replaced in some cases by functional closely related T or D, respectively. The sequence differ mostly in N160, which is replaced by S, A or V. The N160 substitutions could be the main reason for differences in the observed binding ability. (DOCX) [file pone.0159074.s010.docx]

**S2 Table. Alignment of HA sequences of used influenza viruses.** Amino acids involved in binding of PeB as obtained from MD-simulations are highlighted in gray. PDB2vir: sequence obtained from protein database; A/mute/swan/R901/06-H7N1: sequence obtained from Prof. Harder (Friedrich-Loeffler-Institut, Riems, Germany); all other sequences were obtained from influenza virus resource (IVR) as indicated by their accession numbers [62]. While L219 is identical for all subtypes, S159 and E215 are replaced in some cases by functional closely related T or D, respectively. The sequence differ mostly in N160, which is replaced by S, A or V. The N160 substitutions could be the main reason for differences in the observed binding ability.

|  | Positions from 149 till 165 | 149 | 150 | 151 | 152 | 153 | 154 | 155 | 156 | 157 | 158 | **159** | **160** | 161 | 162 | 163 | 164 | 165 |
| --- | --- | --- | --- | --- | --- | --- | --- | --- | --- | --- | --- | --- | --- | --- | --- | --- | --- | --- |
| **Accession number** | **Consensus sequence** | **W** | **T** | **G** | **V** | **T** | **Q** | **N** | **K** | **G** | **G** | **S** | **S** | **A** | **C** | **K** | **R** | **R** |
| Not available | A/mute swan/R901/06(H7N1) | Y | S | . | I | R | T | . | - | . | A | **T** | **.** | . | . | R | . | - |
| CAA24269 | A/Aichi/2/1968(H3N2) | . | . | . | . | . | . | . | - | . | . | **.** | **N** | . | . | . | . | G |
| [AFM71802](http://www.ncbi.nlm.nih.gov/entrez/viewer.fcgi?val=AFM71802) | A/Victoria/210/2009(H3N2) | . | . | . | . | . | . | . | - | . | T | **.** | **.** | . | . | I | . | . |
| ACP44189 | A/California/07/2009(H1N1) | . | P | N | H | D | S | . | . | . | V | **T** | **A** | . | . | P | H | A |
| ABO37541 | A/New York/55/2004(H3N2) | . | . | . | . | . | . | . | - | . | T | **.** | **.** | S | . | . | . | . |
| ABF47955 | A/WSN/1933 TS61(H1N1) | . | P | N | H | . | F | . | - | . | V | **T** | **V** | S | . | S | H | . |
|  |  |  |  |  |  |  |  |  |  |  |  |  |  |  |  |  |  |  |
|  | Positions from 209 till 225 | 209 | 210 | 211 | 212 | 213 | 214 | **215** | 216 | 217 | 218 | **219** | 220 | 221 | 222 | 223 | 224 | 225 |
| **Accession number** | **Consensus sequence** | **H** | **P** | **S** | **T** | **D** | **Q** | **E** | **Q** | **T** | **S** | **L** | **Y** | **A** | **Q** | **A** | **S** | **G** |
| Not available | A/mute swan/R901/06(H7N1) | . | S | G | S | T | T | **.** | . | . | K | **.** | . | G | S | G | N | K |
| CAA24269 | A/Aichi/2/1968(H3N2) | . | . | . | . | N | . | **.** | . | . | . | **.** | . | V | . | . | . | . |
| [AFM71802](http://www.ncbi.nlm.nih.gov/entrez/viewer.fcgi?val=AFM71802) | A/Victoria/210/2009(H3N2) | . | . | V | . | . | K | **D** | . | I | F | **.** | . | . | . | . | . | . |
| ACP44189 | A/California/07/2009(H1N1) | . | . | . | . | S | A | **D** | . | Q | . | **.** | . | Q | N | . | D | A |
| ABO37541 | A/New York/55/2004(H3N2) | . | . | V | . | . | N | **D** | . | I | R | **.** | . | . | . | . | . | . |
| ABF47955 | A/WSN/1933 TS61(H1N1) | . | . | . | S | S | D | **.** | . | Q | . | **.** | . | S | N | G | N | A |
